# The US President's Malaria Initiative, *Plasmodium falciparum* transmission and mortality: A modelling study

**DOI:** 10.1371/journal.pmed.1002448

**Published:** 2017-11-21

**Authors:** Peter Winskill, Hannah C. Slater, Jamie T. Griffin, Azra C. Ghani, Patrick G. T. Walker

**Affiliations:** 1 MRC Centre for Outbreak Analysis and Modelling, Department of Infectious Disease Epidemiology, Imperial College London, London, United Kingdom; 2 School of Mathematical Sciences, Queen Mary University of London, London, United Kingdom; Mahidol-Oxford Tropical Medicine Research Unit, THAILAND

## Abstract

**Background:**

Although significant progress has been made in reducing malaria transmission globally in recent years, a large number of people remain at risk and hence the gains made are fragile. Funding lags well behind amounts needed to protect all those at risk and ongoing contributions from major donors, such as the President’s Malaria Initiative (PMI), are vital to maintain progress and pursue further reductions in burden. We use a mathematical modelling approach to estimate the impact of PMI investments to date in reducing malaria burden and to explore the potential negative impact on malaria burden should a proposed 44% reduction in PMI funding occur.

**Methods and findings:**

We combined an established mathematical model of *Plasmodium falciparum* transmission dynamics with epidemiological, intervention, and PMI-financing data to estimate the contribution PMI has made to malaria control via funding for long-lasting insecticide treated nets (LLINs), indoor residual spraying (IRS), and artemisinin combination therapies (ACTs). We estimate that PMI has prevented 185 million (95% CrI: 138 million, 230 million) malaria cases and saved 940,049 (95% CrI: 545,228, 1.4 million) lives since 2005. If funding is maintained, PMI-funded interventions are estimated to avert a further 162 million (95% CrI: 116 million, 194 million) cases, saving a further 692,589 (95% CrI: 392,694, 955,653) lives between 2017 and 2020. With an estimate of US$94 (95% CrI: US$51, US$166) per Disability Adjusted Life Year (DALY) averted, PMI-funded interventions are highly cost-effective. We also demonstrate the further impact of this investment by reducing caseloads on health systems. If a 44% reduction in PMI funding were to occur, we predict that this loss of direct aid could result in an additional 67 million (95% CrI: 49 million, 82 million) cases and 290,649 (95% CrI: 167,208, 395,263) deaths between 2017 and 2020. We have not modelled indirect impacts of PMI funding (such as health systems strengthening) in this analysis.

**Conclusions:**

Our model estimates that PMI has played a significant role in reducing malaria cases and deaths since its inception. Reductions in funding to PMI could lead to large increases in the number of malaria cases and deaths, damaging global goals of malaria control and elimination.

## Abstract 2

Peter Winskill and colleagues estimate the influence the US President's Malaria Initiative funding has had on averting malaria-related cases and deaths over the past decade and the potential impact if PMI funding is reduced going forward.

## Author summary

**Why was this study done?:**

- The United States contributes a significant proportion of the global budget for malaria control in the form of foreign aid through the President’s Malaria Initiative (PMI).
- Due to proposed cuts to US foreign aid and PMI funding, it is important to demonstrate the impact and cost-effectiveness of PMI.

**What did the researchers do and find?:**

- We used an established malaria transmission model to investigate the impact of PMI funding for malaria control.
- We estimated the past impact of PMI funding on malaria-related cases and deaths and the potential future impact if PMI funding were to be cut.
- PMI funding is highly cost-effective, averting an estimated 185 million cases and saving 940,049 lives since it was set up in 2005.
- A reduction in funding of 44% would lead to an additional 67 million cases and 290,649 deaths over the next 4 years.

**What do these findings mean?:**

- Ongoing support from PMI is critical to maintain recent advances in malaria control and progress towards malaria elimination goals.
- PMI has proven to be a highly cost-effective means by which US foreign aid can be invested to reduce malaria burden.

## Introduction

Unprecedented effort has seen the global burden of malaria halve since the turn of the 21st century due to the widespread distribution of highly effective preventative interventions such as long-lasting insecticide treated nets (LLINs) and indoor residual spraying (IRS) and the provision of highly efficacious treatment with artemisinin combination therapies (ACTs) [1]. However, funding for malaria control has plateaued, falling well behind what is necessary to expand protection to all those in need [2,3]. The continued high level of support for foreign aid contributions in a fluid global political landscape is not guaranteed and gains in malaria control can be fragile if intervention coverage, which is largely dependent on donor funding, is not maintained [4].

The US is the world’s largest donor of foreign aid for malaria control [5] and therefore a mainstay in global malaria efforts. The President’s Malaria Initiative (PMI), established in 2005 and funded by the United States Agency for International Development (USAID), has been particularly influential in investing in malaria control over the past 12 years [6]. PMI provides support to malaria control programmes in 19 African focus countries and the Greater Mekong Subregion (GMS) and is the largest bilateral funder of malaria prevention and treatment [5,7]. In the 12 years since its inception, PMI has procured 197 million LLINs and 378 million courses of ACTs, provided over 215 million person-years of protection with IRS, and distributed 35.7 million courses of preventative therapy for pregnant women [6]. In 2015, PMI funding represented over one-fifth of the global malaria budget envelope [5,6]. In a recent statistical analysis, the influence of PMI funding has been estimated to have had significant impact on under-5 mortality in sub-Saharan Africa, with an estimated reduction of 16% [8]. The US’s commitment to overseas aid has been threatened in recent months [9], highlighting the fragility of global funding for malaria control and a reliance on global political stability. In May 2017, Congress published the Congressional Budget Justification [9], which outlined a commitment to malaria control for 2018 of US$424 million. This is equivalent to a 44% reduction relative to commitments reported for 2017 [10].

To quantify the importance of the PMI contribution to global malaria efforts, we combined data on PMI commodity contributions over time and by country [6] with a mathematical model of the impact of interventions on malaria transmission, morbidity, and mortality parameterised at the subnational level [11] and previously used to inform the Global Technical Strategy (GTS) for malaria [5]. We used this to estimate the global health impact of past PMI funding and the potential implications that a reduction in funding from a key stakeholder and donor in the near term could have on malaria globally.

## Methods

**Fig 1:**
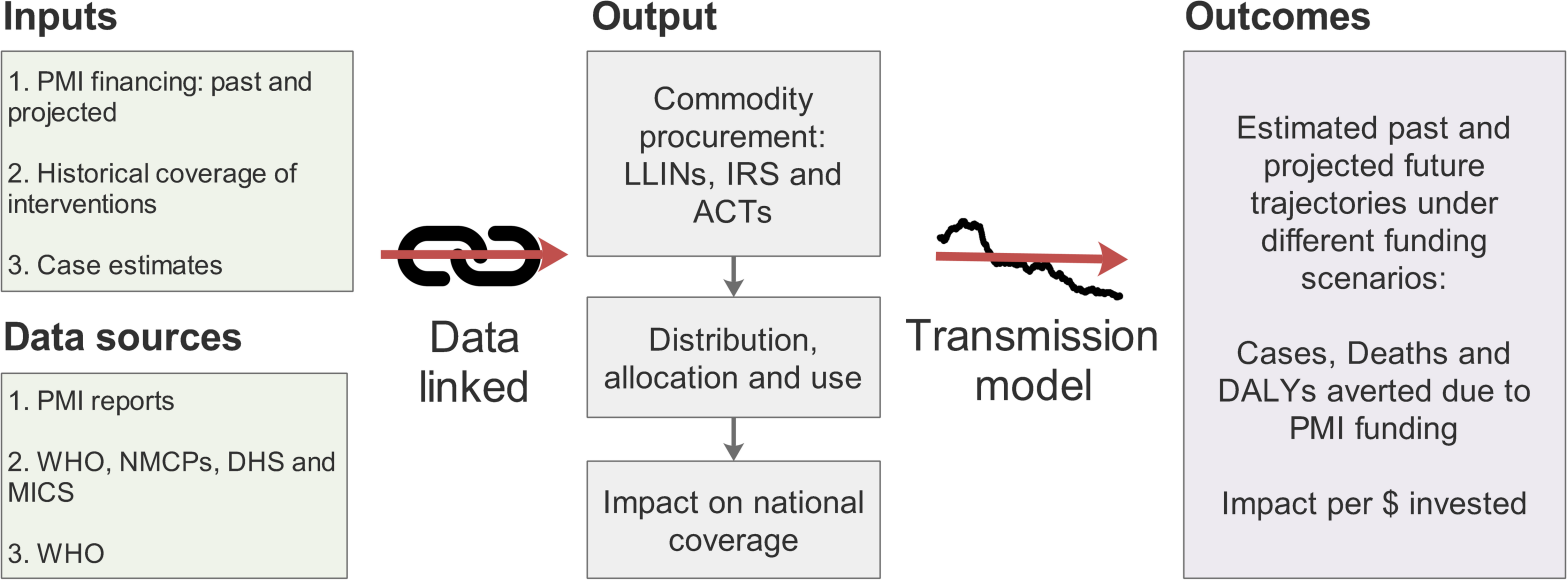
Schematic of the modelling process. Data inputs and sources (left column) are combined and linked to estimate the contribution of PMI and the impact of funding cuts on national-level intervention coverage (middle column). These estimates are then used as inputs in a dynamic transmission model to estimate the impact of changes in intervention coverage on epidemiological outcomes (right column). ACT, artemisinin combination therapy; DALY, Disability Adjusted Life Year; DHS, Demographic and Health Survey; IRS, indoor residual spraying; LLIN, long-lasting insecticide treated net; MICS, Multiple Indicator Cluster Surveys; NMCP, National Malaria Control Programme; PMI, President’s Malaria Initiative.

We linked data on PMI financing, historical intervention coverage, and the underlying epidemiology in modelled countries with estimates of the potential effect of reduction in PMI funding on the coverage of interventions nationally. These estimates were then used as inputs for an established transmission model of *P*. *falciparum* malaria [11,12] to project the impact of reductions in funding on cases, deaths, and Disability Adjusted Life Years (DALYs) (Fig 1).

### Mathematical model

We used an established individual-based malaria transmission model that incorporates a full dynamic mosquito-vector element to allow vector-control interventions to be accurately represented [13]. We briefly describe the model structure below. Full mathematical details can be found in S1 Appendix, Text A-I, and associated references [11,12].

Modelled humans are initially susceptible and may become infected, with a given probability, via the bite of an infectious mosquito. Upon infection, following a period reflecting liver-stage infection, an individual may become symptomatic and seek treatment. Successfully treated individuals benefit from a period of drug-dependent prophylaxis before returning to the susceptible compartment. Symptomatic individuals who do not receive treatment experience a period of symptomatic disease (which has high onward infectivity) before recovering to an asymptomatic state. These individuals, along with those who experience asymptomatic infection, move from being patently asymptomatic to subpatent before natural clearance moves them back into the susceptible compartment. Superinfection can occur from all asymptomatic and subpatent states. Those who experience clinical disease are considered at risk from severe disease and its associated mortality [14].

Naturally acquired immunity is incorporated at several stages of the infection process [12]. Clinical immunity is developed earliest, protecting individuals against severe disease and then clinical disease, and is exposure driven with an age-dependent component to the severe disease pathology and associated mortality rate. Antiparasite immunity develops later, driven by both age and exposure to infection, and reduces the detectability of infections through the control of parasite density. A degree of anti-infection immunity develops later in life, reducing the probability that an infectious bite results in patent infection. The parameters determining the acquisition of immunity were estimated through fitting to severe disease incidence, clinical incidence, and parasite prevalence data stratified by age across a range of transmission settings [12,15].

All infection states are assumed to be onwardly infectious to mosquitoes, with infectivity correlated with parasite density (i.e., highest for clinical disease, intermediate for patent asymptomatic infection, and lowest for subpatent infection), with the parameters estimated by fitting to mosquito feeding studies [12,14,15].

Vectors are modelled as a stochastic compartmental formulation incorporating the larval stages of infection and adult female infection stages [10, 13].

### Geographically specific data inputs

We modelled each first administrative unit (first administrative level below national) in all countries with stable malaria transmission, totalling 1,020 administrative units. Prior scale-up of interventions (LLINs and IRS) was estimated from World Malaria Report data [16], which are based on reports from National Malaria Control Programmes (NMCPs). Demographic and Health Survey (DHS) and Multiple Indicator Cluster Surveys (MICS) for within Africa [17] and World Malaria Report [18] estimates for elsewhere were used to estimate treatment coverage. It was assumed there was no prior scale-up of seasonal malaria chemoprevention (SMC). Each administrative unit was assigned a seasonal pattern that determined the seasonal fluctuation in the carrying capacity of the environment. Seasonality was estimated using Fourier transformations of daily rainfall data from 2002–2009 from Garske et al. (2013) [19]. The carrying capacity was then fitted to 2015 estimates of prevalence (within Africa) [1] or cases (outside of Africa) [16,20] using a root-finding algorithm. Data on populations were compiled from the Gridded Population of the World dataset, adjusted for United Nations estimates of country-level populations [21]. Estimates of the spatial limits of *P*. *falciparum* transmission [20] were used to delimit populations at risk.

### PMI intervention data

To estimate the impact of PMI funding, we firstly estimate the proportion of intervention coverage that is attributable to PMI funding in each location. This is then subtracted from the total intervention coverage estimated. The number of LLINs procured and distributed, the number of people protected by IRS, and the number of ACTs procured and distributed stratified by year and country were all obtained from PMI’s 10th Annual Report to Congress [6]. Absolute numbers were converted to coverage using the appropriate denominators: the estimated population at risk for LLINs and IRS and estimates of the total number of ACT treatment courses delivered [5] for ACT in each country. Examples of this process are detailed in Box 1 (and S1 Table). Throughout, we assumed that 1 LLIN covered 1.8 people (in line with WHO methodology [5]). To estimate the relationships between net delivery, coverage, and usage, we follow an approach by Bhatt et al. (2015) relating distribution data (i.e., procurement as reported by PMI) to household ownership and usage, accounting for household size [22]. The coverage estimates in the model relate to usage and also incorporate wear and tear and decay of insecticide over time. We make an optimistic assumption that ACTs delivered are efficiently used (i.e., reach the health clinics and are effectively employed to treat malaria). In Senegal and Mali, where PMI funds support SMC, we assumed that SMC coverage attributable to PMI was 20%, supporting and complementing SMC implementation by NMCPs and other nongovernmental organisations (NGOs) in these countries [6]. These estimates are then used to simulate malaria trajectories, both retrospectively and prospectively, assuming varying levels of PMI funding.

**Box 1. Example of estimating future coverage attributable to PMI funding: Uganda.**

Estimated population at risk (2015): 37,913,546

**LLINs**:

Number of LLINs distributed (2015) by PMI: 747,320

People covered by LLINs distributed: 747,320 × 1.8 = 1,345,176

LLIN coverage attributable to PMI: 1,345,176 / 37,913,546 = 3.5%

**IRS**:

Number of people protected (2015) by PMI: 3,086,789

IRS coverage attributable to PMI: 3,086,789 / 37,913,546 = 8.1%

**ACTs**:

Number of ACT treatment courses distributed (2015) by PMI: 1,616,130

WHO estimate of total ACT courses delivered: 30,166,620

Estimate of the proportion of treatments attributable to PMI: 1,616,130 / 30,166,620 = 5.3%

### Budget scenarios for 2017 onwards

We considered 3 budget scenarios, one in which PMI funding was kept constant to 2017 levels, one in which 100% of the PMI budget was removed, and a third in which the budget was reduced by 44% (applied uniformly across PMI-supported countries) to reflect the difference in budget attributed to malaria control detailed in the 2017 financial omnibus [10] and the proposed budget for 2018 onwards [9]. The relationship between PMI’s budget and intervention coverage was assumed to be linear, whereby an assumed budget cut of 44% was associated with a proportional decrease in the PMI-attributable intervention coverage. We also ran a scenario with a less drastic reduction in funding of 20%. We assume no mitigation through alternative funding routes or reallocation of reduced budgets. Extra savings and benefits to the health system of PMI funding were also estimated. The savings to the health system of cases averted due to PMI-funded interventions were calculated as the costs of case management and drug commodity costs of the cases averted. In addition, we calculated the additional deaths that may occur if PMI-funded interventions were removed and a national health system did not have the capacity to absorb and adequately treat the additional severe cases.

All scenarios were run multiple times in a sensitivity analysis using 20 separate sets of parameters drawn from the posterior of the modelling fitting [15]. Associated outputs are presented as the median and 95% credible intervals.

## Results

**Fig 2:**
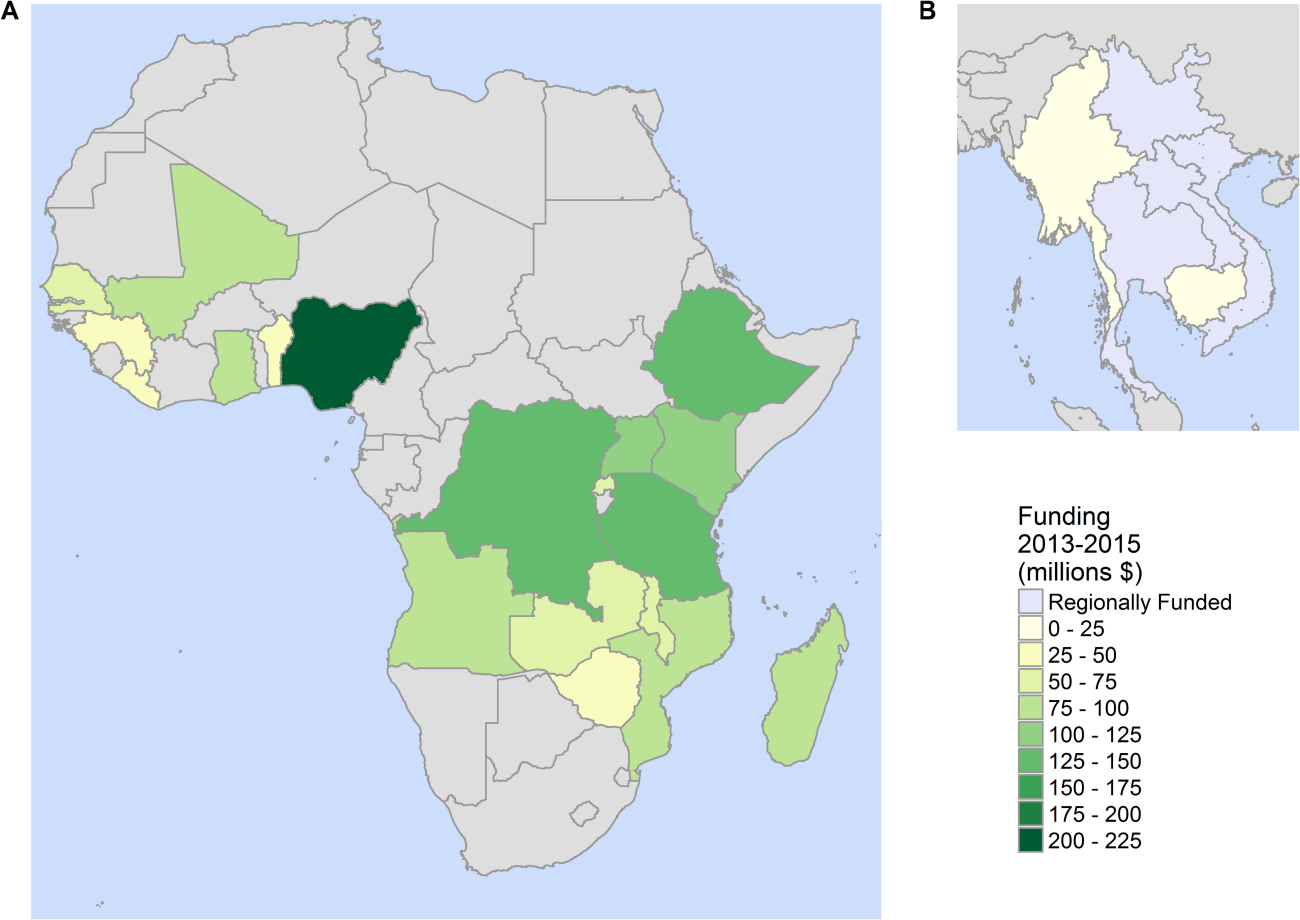
Map of PMI activities. Individual countries and regions that have received PMI-funding and support are highlighted to reflect the level of funding from PMI in (A) sub-Saharan Africa and (B) the GMS over the period 2013–2015. The total regional assignment to the 6 GMS countries over this period is US$9.5 million. Estimated funding per population at risk over this period ranged from US$0.54 (Myanmar) to US$8.08 (Liberia). GMS, Greater Mekong Subregion; PMI, President’s Malaria Initiative.

**Fig 3:**
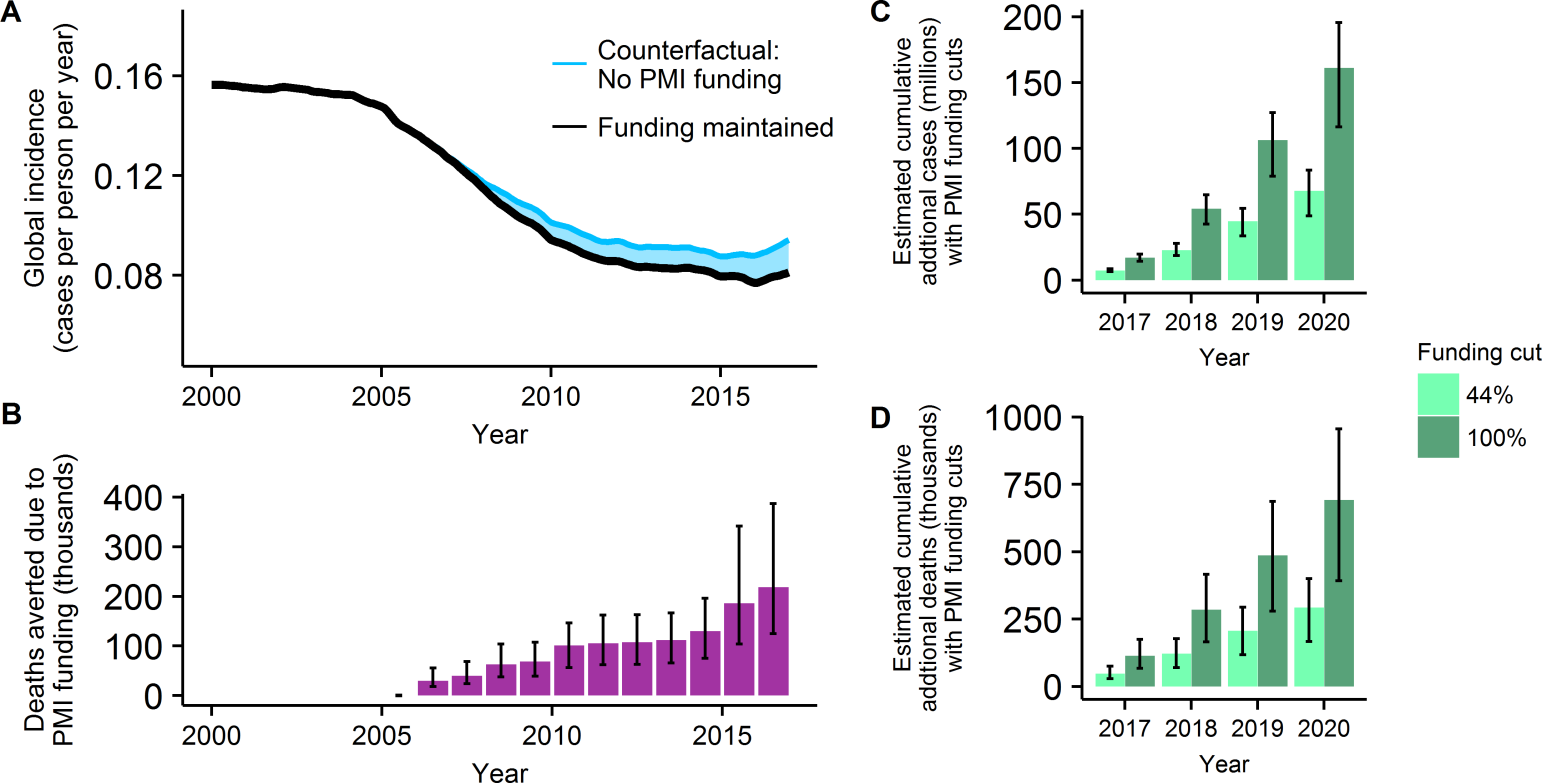
The projected impact of PMI funding on past and future global malaria trends. The (A) past trends (median estimates) in the global incidence of *P*. *falciparum* malaria given funding as occurred (black line) and estimate of the counterfactual trend had PMI support not existed (light blue line). The shaded area represents the cases averted due to PMI funding and (B) shows the associated estimates of death averted each year due to PMI funding. Projected estimates of the additional cumulative numbers of (C) cases (and 95% CrI) and (D) deaths (and 95% CrI) that would occur if PMI funding was reduced by 100% (dark green bars) or 44% (light green bars) over the 4-year period 2017–2020. PMI, President’s Malaria Initiative.

To date, PMI has allocated over US$5 billion to 19 PMI focus countries in sub-Saharan Africa as well as the GMS [23] (Fig 2). We attribute increases in coverage of 8.13% for LLINs, 4.18% for IRS, and 12.9% for ACTs to PMI funding in supported countries in 2015. We estimate that in the 12 years since its inception, PMI has prevented 185 million malaria cases (95% CrI: 138 million, 230 million) (Fig 3A) and saved 940,049 lives (95% CrI: 545,228, 1.4 million) (Fig 3B), the majority of which (77%, 95% CrI: 75%, 81%) would have occurred in children under the age of 5. In sub-Saharan Africa, we estimate that PMI investment has led to an 11.6% (95% CrI: 9.5%, 13.0%) reduction in incidence and an 18.3% (95% CrI: 16.3%, 20.4%) reduction in under-5 malaria-mortality rates in 2015. We estimate the biggest impact in terms of absolute cases averted to have occurred in long-term supported countries with the highest burden. For example, Nigeria, the country with the highest burden globally [5], has received approximately US$345 million from PMI since 2010 [6], leading to an estimated 13.8 million cases (95% CrI: 8.7 million, 17.0 million) averted and 128,861 lives (95% CrI: 75,852, 200,075) saved. Angola has benefitted from continuous support since 2005, seeing investments totalling US$248 million dollars [6], leading to an estimated 8.7 million cases (95% CrI: 6.3 million, 10.4 million) averted and 43,752 lives (95% CrI: 24,946, 61,433) saved.

We estimate that a 44% cut in PMI funding would lead to an additional 67 million cases (95% CrI: 49 million, 82 million) (Fig 3B) and 290,649 deaths (95% CrI: 167,208, 395,263) (Fig 3C; S1 Appendix, Table F) from malaria compared to maintaining current levels of funding from 2017 to 2020. A 20% reduction in funding was associated with an additional 31 million cases (95% CrI: 21 million, 38 million) and 127,799 deaths (95% CrI: 73,313, 178,234) over the same period. If PMI-funded coverage of interventions can be maintained over the next 4 years, PMI support will be responsible for averting an estimated total of 162 million cases (95% CrI: 116 million, 194 million) (Fig 3B) and 692,589 deaths (95% CrI: 392,694, 955,653) (Fig 3C) in the 4-year period from 2017 to 2020, compared to no PMI support.

The impact on malaria burden will be focussed in high-burden countries receiving significant financial support in sub-Saharan Africa. Additionally, with ongoing concern surrounding the emergence and spread of ACT drug resistance [24], support for the GMS is also contributing to the malaria elimination goals in that region.

**Fig 4:**
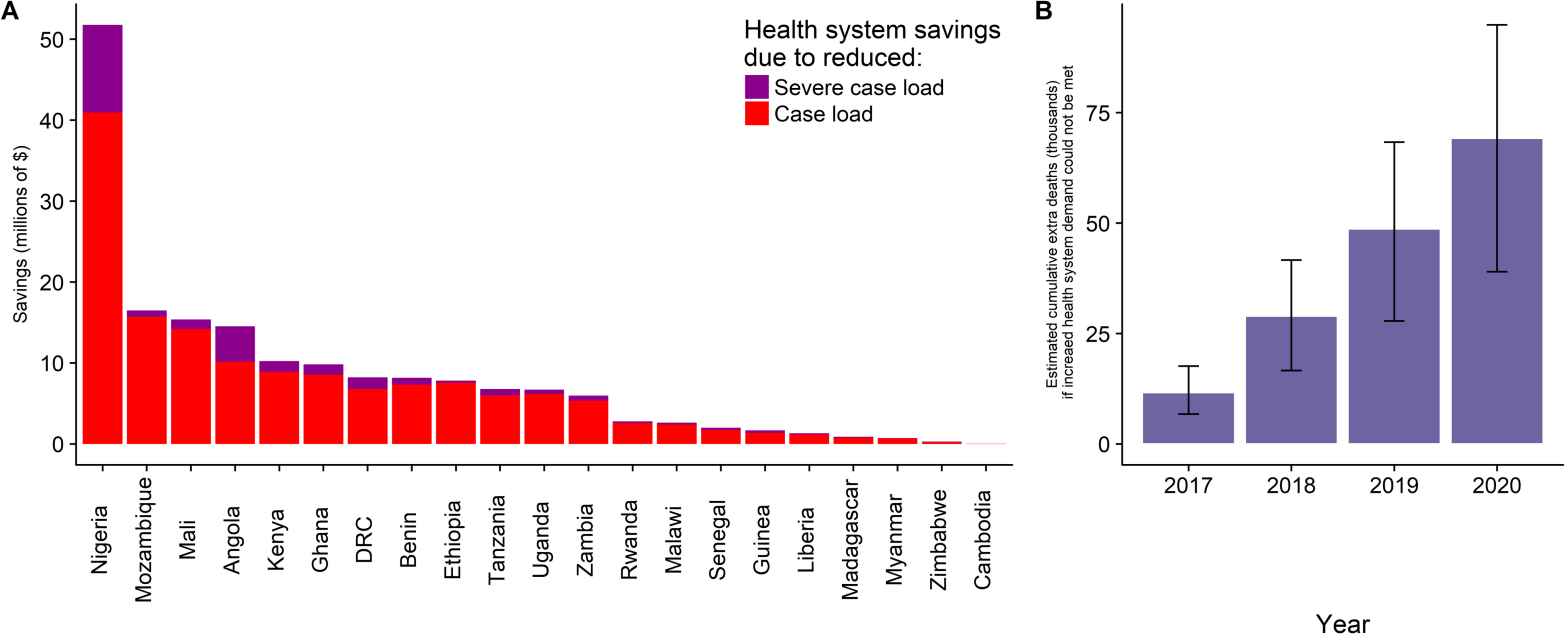
The health-system benefits associated with PMI funding. PMI investment in malaria interventions reduces caseloads of national health systems with resulting (A) averted spending due to reduced treatments of clinical and severe cases by country. Without PMI investment, these health system gains are lost, potentially resulting in (B) the estimated cumulative malaria-related deaths in addition to those caused directly by removal of interventions due to health systems not being able to respond to increased caseloads. PMI, President’s Malaria Initiative.

We estimate that PMI support would avert an additional US$174 million dollars (95% CrI: 121 million, 224 million) of national health system spending through averted malaria cases from 2017 to 2020 (Fig 4A). In the absence of PMI funding, a failure of health systems to absorb the extra caseload (through lack of capacity, finances, or both) would lead to an estimated 69,314 extra deaths (95% CrI: 39,102, 94,888) over this period (Fig 4B), in addition to the 692,589 deaths estimated to be directly caused by reductions in intervention coverage. These impact estimates are likely conservative, not accounting for the indirect impacts of increased transmission.

Over the period 2013–2015, when the PMI programme was fully scaled to current levels, PMI reported that spending in the 19 focus countries in sub-Saharan Africa was approximately US$1.7 billion. Translating the modelled epidemiological impact into system-wide cost-effectiveness, we estimate a cost of US$20.6 per malaria case averted (95% CrI: US$15.2, US$31.4), US$4,081 per death averted (95% CrI: US$2,084, US$7,435), and US$94 per DALY averted (95% CrI: US$51, US$166) (Table 1). This represents a range of 2%–57% as a proportion of per-capita gross domestic product (GDP) in these countries. Cost-effectiveness estimates are driven by the intervention mix and national-level differences in the cost of treating clinical and severe cases. Differences between cases and deaths averted are primarily driven by the intervention mix, especially the proportion of funding that went towards treatment (treatment contribution is positively associated with the proportion of deaths to cases averted, linear model coefficient = 0.012, *p* = 0.035).

**Table 1:** Summary of PMI support and estimated impact for 19 focus countries (2013–2015).

Country	Funding (US$ millions) [6]	People protected by IRS (millions) [6]	LLINs distributed (millions) [6]	ACTs distributed (millions) [6]	Cases averted (millions)	Deaths averted (thousands)	DALYs averted (millions)	Cost per case averted	Cost per death averted	Cost per DALY averted
**Angola**	85.55	1.15	2.71	6.55	2.9 (2, 3.4)	13.3 (7.6, 19.2)	0.6 (0.3, 0.9)	30 (42, 25)	6,431 (4,445, 11,276)	144 (98, 253)
**Benin**	49.65	2.29	2.65	2.46	2.7 (1.9, 4)	11.3 (6.5, 22.1)	0.6 (0.3, 1.1)	18 (26, 12)	4,394 (2,251, 7,625)	87 (44, 147)
**DRC**	141.87	0	2.15	17.85	3.3 (1.9, 5.2)	35.8 (21.1, 54)	1.8 (1.1, 2.8)	43 (73, 27)	3,966 (2,626, 6,724)	81 (52, 135)
**Ethiopia**	132.77	4.94	10.71	7.22	10.4 (5, 18.4)	73.6 (30.1, 233.1)	1.8 (0.7, 5.6)	13 (27, 7)	1,805 (570, 4,415)	74 (24, 187)
**Ghana**	84.55	1.66	5.63	6.28	4.3 (2.3, 5.8)	19.4 (11.5, 29)	1 (0.6, 1.5)	19 (36, 14)	4,355 (2,910, 7,371)	87 (57, 142)
**Guinea**	37.37	0	1.48	2.83	1 (0.5, 1.3)	9.8 (6.8, 20.9)	0.5 (0.3, 1.1)	39 (71, 28)	3,808 (1,789, 5,533)	74 (36, 107)
**Kenya**	104.26	0	4.46	26.86	4.1 (2.9, 5)	21.4 (12.3, 30.9)	1 (0.5, 1.4)	25 (36, 21)	4,861 (3,371, 8,504)	108 (76, 193)
**Liberia**	36.37	0.37	0.31	4.56	0.3 (−0.1, 0.8)	2.5 (1.1, 3.9)	0.1 (0.1, 0.2)	117 (−404, 47)	14,496 (9,428, 31,639)	262 (179, 534)
**Madagascar**	78.03	5.14	2.32	1.86	5.9 (4.4, 6.9)	31.9 (16.9, 51.6)	1.3 (0.7, 2.1)	13 (18, 11)	2,446 (1,511, 4,621)	61 (37, 114)
**Malawi**	68.08	0	2.02	17.32	2.6 (1.9, 3.2)	12.1 (7.3, 16.2)	0.6 (0.4, 0.9)	27 (35, 22)	5,643 (4,192, 9,347)	108 (80, 178)
**Mali**	75.01	2.18	5.55	6.29	5.9 (4.6, 7.6)	20 (12.3, 59.8)	1 (0.6, 3.1)	13 (16, 10)	3,743 (1,255, 6,109)	73 (24, 119)
**Mozambique**	87.02	7.23	4.1	24.48	6.1 (3.6, 8.5)	20.3 (12.6, 45.9)	1.1 (0.7, 2.4)	14 (24, 10)	4,284 (1,897, 6,887)	80 (36, 129)
**Nigeria**	223.27	0.35	13.87	27.7	11.6 (7.6, 14.3)	73.4 (44.5, 113)	3.6 (2.1, 5.6)	19 (30, 16)	3,044 (1,976, 5,014)	62 (40, 104)
**Rwanda**	53.5	2.94	2	2.45	2.4 (1.7, 3)	6.6 (4.1, 9.4)	0.3 (0.2, 0.4)	23 (32, 18)	8,063 (5,674, 13,008)	173 (122, 281)
**Senegal**	72.12	1.91	1.6	1.23	3 (2.2, 3.7)	8.5 (4.9, 11.8)	0.4 (0.2, 0.6)	24 (33, 19)	8,463 (6,101, 14,722)	184 (129, 318)
**Tanzania**	138.06	9.85	2.24	12.4	6.1 (4.7, 7.5)	28.5 (15.1, 47.9)	1 (0.5, 1.7)	23 (30, 18)	4,839 (2,881, 9,133)	136 (80, 255)
**Uganda**	101.78	8.23	1.82	2.71	2.8 (1.7, 4.2)	6.4 (2.2, 10.2)	0.4 (0.2, 0.6)	36 (59, 24)	16,015 (10,004, 45,535)	264 (179, 648)
**Zambia**	72.03	5.63	2.54	11.73	5.2 (3.7, 6.8)	12 (6.5, 19.8)	0.7 (0.4, 1.1)	14 (19, 11)	6,006 (3,637, 11,017)	99 (65, 171)
**Zimbabwe**	45.04	2.93	1.45	2.83	1.2 (1.1, 1.4)	6.4 (3.4, 10.2)	0.2 (0.1, 0.4)	37 (43, 32)	7,059 (4,403, 13,276)	193 (120, 374)
**Total/Summary**	1,686.32	56.80	69.6	185.6	81.8 (53.6, 11.0)	413.2 (226.8, 808.9)	18.0 (10.2, 33.4)	20.6 (15.2, 31.4)	4,081 (2,084, 7,435)	93.8 (50.5, 166.0)

**Abbreviations:** ACT, artemisinin combination therapy; DALY, Disability Adjusted Life Year; DRC, Democratic Republic of the Congo; IRS, indoor residual spraying; LLIN, long-lasting insecticide treated net; PMI, President’s Malaria Initiative.

## Discussion

Here, we have produced modelled estimates of the programme-wide effectiveness of PMI in terms of the impact it has had upon malaria morbidity and mortality since its inception in 2005. We estimate that PMI has averted 185 million cases and 940,049 deaths to date. If funding for PMI is maintained, we predict that a further 162 million cases and 692,589 deaths could be averted over the next 4 years, compared to no PMI funding. However, in comparison to continued full PMI support, a 44% cut in the PMI budget, as indicated in the May 2017 Congressional Budget Justification, could result in an additional 67 million cases and 290,649 deaths in the next 4 years.

Our results highlight the fragility of the gains in malaria control that have been made to date, particularly given the changing geopolitical landscape [25]. International funding, including that from governments, such as from PMI, the United Kingdom’s Department for International Development (DFID), the Global Fund, and others, accounts for a large proportion (approximately 68% [5]) of the funds available for malaria control worldwide. Malaria control is therefore reliant on sustained long-term investment from foreign donors. Without continued commitment to support programmes, recent gains in the control of malaria will be difficult to sustain and potential rebound epidemics likely [4].

Prudent investment of foreign aid relies on being able to effectively implement cost-effective interventions to maximise health gains. PMI has proven to be a capable mediator of this process for malaria. The estimates of cost per DALY averted here are significantly below the WHO threshold for cost-effectiveness of less than 300% of a country’s per-capita GDP [26]. Even among highly cost-effective interventions, malaria control compares favourably as a means by which to improve global health [27]. Between-country variation in cost-effectiveness is pronounced. The effect is driven by the intervention mix and underlying epidemiological variation (such as the intrinsic transmission potential). Costs are driven by the intervention mix and, specifically, the impact of PMI support on treatment costs, which varies between countries. Whilst the past and current positive health impacts of PMI-funded interventions is very apparent, there remains much debate as to the impact that foreign aid has on recipient countries [28].

In addition to its direct impact on cases and malaria-attributable mortality, investment in malaria control brings about substantial further potential health gains by alleviating the burden that malaria places on health systems in affected countries [29]. Supporting vector control interventions is expected to decrease caseloads, freeing up health system capacity and reducing costs incurred from treating clinical and severe cases of malaria. A recent PMI-supported study demonstrated reductions in malaria-related inpatient and outpatient admissions and hospital costs after the scale-up of interventions in Southern Province in Zambia [30]. Funding cuts lead to increased caseloads due to the negative impacts of reduced intervention coverage, the stress of which will be borne by the national health systems of malaria-endemic countries. Lack of health-system capacity was a critical factor in the recent Ebola epidemic in West Africa [31], the impact of which reverberated globally. Those countries worst affected are highly malaria endemic and had health systems already dealing with the challenges of a high malaria burden [32,33]. A redistribution of emergency funds earmarked for the Ebola epidemic [9] could potentially help to mitigate budget cuts for malaria control. However, this is a finite fund that would only serve as a very near-term solution to budget reductions.

Our results provide a conservative estimate of the overall impact of PMI funding, as we do not capture the impact of all PMI-associated activities, notably intermittent preventive treatment in pregnancy (IPTp), which we have not modelled but is one of the most cost-effective malaria interventions [34,35]. PMI presence in a country further catalyses and facilitates the procurement, distribution, and implementation of interventions from other funders with the initiative distributing 80 million LLINs and 34 million ACT courses procured by other donors in the period 2006–2015 [6]. Furthermore, PMI is involved with a number of capacity and health system-strengthening initiatives, such as training health workers in malaria diagnosis and treatment [6], the loss of which would compound issues of increased caseload if PMI support were reduced. Our estimates of reductions in under-5 mortality attributable to PMI funding are lower when compared with estimated reductions of a similar magnitude in all-cause mortality in a recently published difference-in-differences analysis of PMI impact [7]. Whilst our estimates of intervention coverage attributable to PMI funding are similar, the additional impact estimated by Jakubowski et al. may be ascribed to indirect impacts of PMI funding on nonmalaria outcomes (through, for example, health systems strengthening), although considerable uncertainties also impact both analyses. We also do not capture the wider societal costs of the disease, such as missed workdays by carers, reduced education, or impact on future lifetime earnings, nor the economic effects of endemic malaria on factors such as migration, trade, tourism, or foreign investment within a country [36]. It is likely that, when facing cuts, PMI and NMCPs would reallocate existing funds to cover those interventions seen as vital. However, in an already budget-restricted environment, a limit to the potentially mitigating effects of such reallocations would quickly be reached. There are a number of difficulties associated with estimating accurate coverage estimates and uptake for interventions with a wide range of definitions and methodologies adopted. We have assumed that PMI-reported contribution and interventions figures, taken from their 10th Annual Report to Congress [6] and building upon a well-established monitoring and evaluation strategy, are representative and accurate. We also are including assumptions that the PMI-delivered interventions are reaching required recipients in an efficient manner. Whilst we know inefficiencies do exist, for example in LLIN distribution [22], these are difficult to attribute to specific sources. Furthermore, due to the nonlinear impact of interventions such as LLINs, it is difficult to split contributions from different funding sources (i.e., should an X% funding contribution be linked to the first N% or last N% of observed LLIN coverage?). We do account for falloff between coverage and usage as well as deterioration of insecticide and wear and tear of LLINs in this analysis. Similarities to empirical estimates [8] indicate that we are accurately capturing broad trends in intervention coverage due to PMI funding.

As malaria transmission is brought to low levels, increased efforts are needed to target hard-to-reach populations as well as increase surveillance efforts, and hence the programmatic costs are likely to increase [7]. In such circumstances, investment decisions need to take into account the potential for permanent gains that would be accrued if an area or country can achieve elimination. However, there still remain large, extremely cost-effective gains that can be obtained by investing further to reduce the burden of malaria in areas of high endemicity. WHO GTS for malaria has set targets of achieving of at least 90% reductions in global case incidence and mortality rates by 2030 compared to levels in 2015, with vector control, chemoprevention, diagnosis and treatment, and surveillance being key pillars of the outlined strategy [37]. Based on the estimates of our model, PMI’s ongoing support of these activities in countries of high burden or strategic importance is vital in order to avoid a rapid erosion of the progress made in the last 15 years on the road towards malaria eradication.

## Supporting information

S1 Appendix Model details, site characterisation, and further methodology. (DOCX)

S1 Table PMI-attributable coverage estimation. PMI, President’s Malaria Initiative.
(XLSX)

S2 Table First administrative unit parameterisation. (XLSX)

## Abbreviations

ACT: artemisinin combination therapy
DALY: Disability Adjusted Life Year
DFID: Department for International Development
DHS: Demographic and Health Survey
GDP: gross domestic product
GMS: Greater Mekong Subregion
GTS: Global Technical Strategy
IPTp: intermittent preventive treatment in pregnancy
IRS: indoor residual spraying
LLIN: long-lasting insecticide treated net
MICS: Multiple Indicator Cluster Surveys
NGO: nongovernmental organisation
NMCP: National Malaria Control Programme
PMI: President’s Malaria Initiative
SMC: seasonal malaria chemoprevention
USAID: United States Agency for International Development
