# Supplementary material for: The US President's Malaria Initiative, Plasmodium falciparum transmission and mortality: A modelling study
Source: PLoS Med. 2017 Nov 21;14(11):e1002448. doi: 10.1371/journal.pmed.1002448 (PMC5697814; doi:10.1371/journal.pmed.1002448)
Supplement: S1 Appendix — (DOCX) [file pmed.1002448.s001.docx]

Supplementary Information

# Text A. Transmission Model

We use a previously published malaria transmission model that incorporates the full dynamics of Plasmodium falciparum transmission between human and vector hosts. The model is similar to that presented in Griffin et al. 2016 [1] with some recent modification to the LLIN/IRS model as detailed here. For completeness, in the following sections we give the full model details and all parameters used in this analysis. The model has previously been extensively fitted to data on the relationship between vector density, EIR, parasite prevalence, uncomplicated malaria, severe disease and death [1–5].

## Text B. The Human Component

Individuals begin life susceptible to infection (*S*). New-born individuals possess a level of maternally-inherited immunity that decays over the first six months of their lives. Throughout their life, individuals are exposed to infectious bites from the mosquito vector. The hazard of infection is determined by the force of infection acting on individual *i*,$\left(\Lambda_{i}\right)$. This force of infection is determined by the individual’s level of pre-erythrocytic immunity, biting rate and the mosquito population size and level of infectivity. Following a latent period $\left(d_{E}\right)$, infected individuals may either develop clinical disease or asymptomatic infection (moving to infection state *A*). The outcome of infection is determined by the individual’s probability of acquiring clinical disease which is dependent on their level of clinical immunity. Individuals who develop clinical disease be successfully treated (with a fixed probability $f_{T}$), moving to infection state *T*, or will not seek treatment (with probability $1-f_{T}$) moving to infection state *D*. Treated individuals then recover from infection at rate $r_{T}$ and return to the susceptible infection state *S*. However, they retain a degree of drug-dependent partial protection from reinfection (modelled as a Weibull survivorship curve) which wanes over time [6]. Individuals not receiving treatment recover to the asymptomatic infection state (*A*) at rate$r_{D}$. As parasite density is controlled, asymptomatic individuals progress to the subpatent infection state (*U*) with rate$r_{A}$, before naturally clearing infection, returning to susceptible (*S*) with rate$r_{U}$. Superinfection is included with all individuals in states *D*, *A* and *U* remaining susceptible to re-infection. If this occurs, the individuals move into infection states *D*, *T* or *A* in the same process described above.


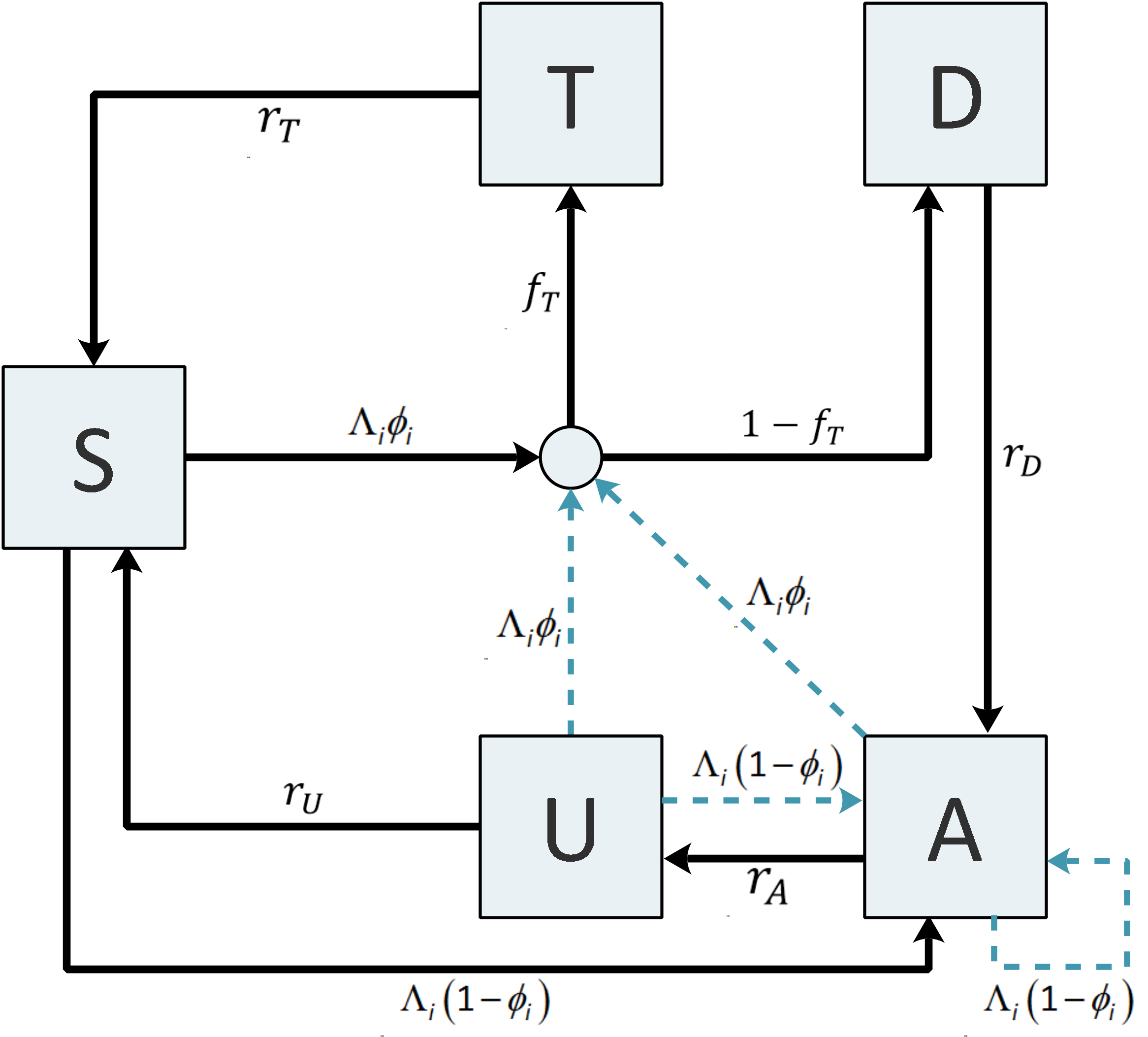


Figure A. Illustration of the progression between human infection states. States are shown in boxes and state transitions by arrows with associated hazard rates. The circle represents the treatment node. Superinfection is indicated by dashed blue arrows. *S* = susceptible, *D* = clinical disease*, T* = successfully treated disease*, A* = asymptomatic patent infection*, U* = asymptomatic subpatent infection.

Death from natural causes is modelled using national life-tables [7], with individuals removed from the population at age-specific rates to match the required age distribution. Malaria-associated deaths are tracked separately (see Section 1.1.4). When an individual dies they are replaced with a new-born individual with the same heterogeneity level in exposure to infection so that the population size in the simulation remains constant. The infection progression pathway is illustrated in Fig. A.

The rates associated with the state transitions are summarised in Table A.

Table A: The rates associated with transitions between infection states for the human model.

| Process | Transition | Rate |
| --- | --- | --- |
| Infection | $S\to I$ | $\Lambda_{i}\left(t-d_{E}\right)$ |
| Progression of untreated disease to asymptomatic infection | $D\to A$ | $r_{D}=\frac{1}{d_{D}}$ |
| Progression of asymptomatic to subpatent infection | $A\to U$ | $r_{A}=\frac{1}{d_{A}}$ |
| Progression of subpatent infection to susceptible | $U\to S$ | $r_{U}=\frac{1}{d_{U}}$ |
| Progression of treated disease to susceptible* | $T\to S$ | $r_{T}=\frac{1}{d_{T}}$ |
| Super-infection from untreated clinical disease, asymptomatic or subpatent infection | $D\to I$ $A\to I$ $U\to I$ | $\Lambda_{i}\left(t-d_{E}\right)$ |

* Treated individuals experience a period of drug-dependent partial protection from reinfection.

### Text C. Biting Rate

Individuals are assigned a unique biting rate which is the product of their relative age-dependent biting rate, $\psi_{i}$, which, for age *a*, is defined as:

$$\psi\left(a\right)=1-\rho\exp\left(-\frac{a}{a_{0}}\right)$$

where $\rho$ and $a_{0}$ are parameters that determine the relationship between age (i.e. body size) and biting rate and$\omega$ is a normalising constant for the biting rate with age:

$$\omega=\int_{0}^{\infty} \psi(a)g(a) da$$

where $g(a)$ is the cross-sectional human population age distribution. The relative biting rate is drawn from a Log-normal distribution with a mean of 1:

$\log\left(\zeta_{i}\right)\sim N\left(\frac{-\sigma^{2}}{2},\sigma^{2}\right)$ .

The EIR $\varepsilon_{i}(a,t)$ and force of infection $\Lambda_{i}(a,t)$ experienced by individual *i* with age *a* at time *t* can be denoted as:

where $\varepsilon_{0}(t)$ is the mean EIR experienced by adults at time *t* and $b_{i}(t)$is the probability that an infectious bite leads to a patent infection, which is determined by the level of pre-erythrocytic immunity (see Section 1.1.2). This is then subject to a lag of $d_{E}$ days to account for the latent period of infection.

### Text D. Immunity

The acquisition and loss of naturally-acquired immunity is captured dynamically in the model, driven by both age and exposure as detailed here. Infants acquire a level of maternal immunity to clinical disease and severe disease at birth, denoted $I_{CM}$ and $I_{VM}$ respectively. The level at birth is set as a proportion, $P_{M}$, of the acquired immunity of a randomly chosen 15-35 year-old in the population with the same heterogeneity level. This decays exponentially at a constant rate $r_{M}=1/d_{M}$.

Blood-stage immunity is tracked through its impact on three outcomes: i) the probability of developing severe disease, ii) the probability of developing clinical disease and iii) the detectability of asymptomatic infection – which develop in this order through the associated parameterisations below. Acquired immunity to severe disease, clinical disease and detectability of infection is boosted by one level following each patent infection provided it is at least $u_{V}$, $u_{C}$ or $u_{D}$days respectively since the last exposure, and decays exponentially between exposures with rate $r_{VA}=1/d_{VA}$,$r_{CA}=1/d_{CA}$and $r_{ID}=1/d_{ID}$respectively. Acquired immunity to infection (pre-erythrocytic immunity) develops at a later age in a similar manner, but is boosted by one level following each infected bite rather than patent infection provided it is at least $u_{B}$ days since the last exposure, and decays exponentially in between exposures with rate $r_{B}=1/d_{B}$. It reduces the probability of developing a patent infection following an infectious bite.

Immunity levels are converted to individual time-dependent probabilities using Hill functions. The probability that individual *i* who is exposed to an infectious bite at time *t* develops a patent infection is given by:

$$b_{i}(t)=b_{0}\left(b_{1}+\frac{1-b_{1}}{1+\left(\frac{I_{B}(i,t)}{\mathrm{I}_{B0}}\right)^{\kappa_{B}}}\right)$$

where $b_{0}$ is the probability of infection with no immunity, $b_{0}b_{1}$ is the minimum probability, $I_{B0}$ and $\kappa_{B}$ are scale and shape parameters respectively and $I_{B}(i,t)$ is the level of pre-erythrocytic immunity of individual *i* at time *t*.

The probability that individual *i* develops clinical disease at time *t* upon being infected is defined as:

where is the probability of disease with no immunity, is the minimum probability, $I_{C0}$ and $\kappa_{C}$ are scale and shape parameters respectively, $I_{CA}(i,t)$is the level of acquired immunity to clinical disease and $I_{CM}(i,t)$ is the level of maternally acquired immunity to clinical disease of individual *i* at time *t*.

The probability that individual *i* develops severe disease at time *t* and age *a* upon being infected is defined as:

$$\theta_{i}(a,t)=\theta_{0}\left(\theta_{1}+\frac{1-\theta_{1}}{1+f_{V}\left(i,a\right)\left(\frac{(I_{VA}\left(i,t\right)+I_{VM}\left(i,t\right)}{I_{V0}}\right)^{\kappa_{V}}}\right)$$

where $\theta_{0}$ is the probability of disease with no immunity, $\theta_{0}\theta_{1}$ is the minimum probability, $I_{V0}$ and $\kappa_{V}$ are scale and shape parameters respectively, $I_{VA}(i,t)$is the level of acquired immunity to severe disease, $I_{VM}(i,t)$ is the level of maternally acquired immunity to severe disease of individual *i* at time *t* and

$$f_{V}(i,a)=1-\frac{\left(1-f_{V0}\right)}{\left(1+\left(\frac{a}{a_{V}}\right)^{\gamma_{V}}\right)}$$

is an age-dependent (physiological) modifier of the risk of severe disease, where $f_{V0}, \mathrm{a}_{V}$ and $\gamma_{V}$ are parameters.

The detectability by microscopy of an asymptomatic infection in individual *i* of age *a* at time *t*  is given by:

$$q_{i}(a,t)=d_{1}+\frac{(1-d_{\min})}{\left(\left(\frac{1+I_{D}\left(i,t\right)}{I_{D0}}\right)^{\kappa_{D}}f_{D}\left(i,a\right)\right)}$$

where $d_{\min}$ is the minimum probability of detection, $I_{D0}$ and $\kappa_{D}$ are scale and shape parameters respectively, $I_{D}(i,t)$ is the level of acquired immunity to the detectability of infection of individual *i* at time *t* and

$$f_{D}(i,a)=1-\frac{\left(1-f_{D0}\right)}{\left(1+\left(\frac{a}{a_{D}}\right)^{\gamma_{D}}\right)}$$

is an age-dependent (physiological) modifier of the detectability of infection where $f_{D0}, \mathrm{a}_{D}$ and $\gamma_{D}$ are parameters.

### Text E. Infectivity to mosquitoes

The lower parasite density which reduces the probability of detection also is assumed to decrease the probability of onwards transmission to the mosquito. Onwards infectiousness is $c_{D}$and $c_{U}$ in states $D$and $U$respectively, and $c_{T}$following treatment. In state $A$ infectiousness is modified by $q$, being defined as $c_{U}+(c_{D}+c_{U})\mathrm{q}^{\gamma_{I}}$.

### Text F. Severe Disease and Mortality

Severe disease and malaria-associated mortality are derived from the population outputs from the model. Following Griffin *et al* (2016)[1], the incidence of severe malaria requiring hospitalisation in the age range $a_{L}$ to $a_{U}$ at time *t* is given by:

$$\lambda_{H}\left(t,(a_{L},a_{U})\right)=\frac{\sum_{i : a_{L}< a_{i}(t) <a_{U}} \left((1-f_{T})+f_{T}f_{VT}\right)\Lambda_{i}(t) \theta_{i}(t)}{\#\left\{i : a_{L}< a_{i}(t) <a_{U}\right\}}$$

where $\Lambda_{i}(t)$ is the force of infection experienced by individual *i* at time *t* and $\theta_{i}(t)$ the probability that individual *i* develops severe disease at time *t* upon being infected . Malaria-related mortality is assumed to be in proportion to the incidence of severe disease due to malaria and is defined as:

$$\mu(t,(a_{L},a_{U}))=v\lambda_{H}\left(t,(a_{L},a_{U})\right)$$

where parameter $v$ is a scaling factor, estimated to be 0.215 [1]. Individuals receiving treatment are assumed to experience a reduction, $f_{VT}$, in the probability of disease progression to severe disease and hence death [1]. Considering the scarcity of data to inform this parameter, we assumed a nominal value of 0.5.

### Text G. Stochastic Equations

The full stochastic individual-based human component of the model can be formally described by its Kolmogorov forward equations. Let $i$ index individuals in the population. Then the state of individual $i$ at time $t$ is given by $\{j,t_{T},k,t_{k},l,t_{l},m,t_{m},n,t_{n,}a,t\}$, where $a$ is age, $j$ represents infection status $(S,D,A,U,T)$, $t_{T}$ is the time at which individual $i$ was last treated, *k* is their level of infection-blocking immunity and $t_{k}$ is the time at which infection blocking immunity was last boosted. Similarly, *l* and $t_{l}$ denote the level and time of last boosting of naturally-acquired clinical immunity respectively, *m* and $t_{m}$ do likewise for parasite detection immunity, and *n* and $t_{n}$for severe disease immunity. Maternally-acquired immunity levels to clinical disease and severe diseases are denoted by $l_{mat}$ and $n_{mat}$respectively.

Using standard mathematical notation, let $\delta_{p,q}$ denote the Kronecker delta ($\delta_{p,q}=1$ if *p=q* and 0 otherwise) and $\delta(x)$ denote the Dirac delta function.

We define $P_{i}(j,t_{T},k,t_{k},l_{mat},l,t_{l},m,t_{m},n_{mat},n,t_{n},a,t)$ as the probability density function for individual $i$ being in state $\{j,t_{T},k,t_{k},l_{mat},l,t_{l},m,t_{m},n_{mat},n,t_{n,}a,t\}$ at time $t$. The time evolution of the system is governed by the following forward equation:

| **1** | $\frac{\partial P_{i}(j,t_{T},k,t_{k},l_{mat},l,t_{l},m,t_{m},n_{mat},n,t_{n},a,t)}{\partial t}+\frac{\partial P_{i}(j,t_{T},k,t_{k},l_{mat},l,t_{l},m,t_{m},n_{mat},n,t_{n},a,t)}{\partial a}=$ |
| --- | --- |
| **2** | $\delta_{j,S}[r_{T}P_{i}(T,t_{T},k,t_{k},l_{mat},l,t_{l},m,t_{m},n_{mat},n,t_{n},a,t)+r_{U}P_{i}(U,t_{T},k,t_{k},l_{mat},l,t_{l},m,t_{m},n_{mat},n,t_{n},a,t)]+$ |
| **4** | $\delta_{j,A}[r_{D}P_{i}(D,t_{T},k,t_{k},l_{mat},l,t_{l},m,t_{m},n_{mat},n,t_{n},a,t)]+$ |
| **5** | $\delta_{j,U}[r_{A}P_{i}(A,t_{T},k,t_{k},l_{mat},l,t_{l},m,t_{m},n_{mat},n,t_{n},a,t)]+$ |
| **6** | $(1-b_{i})\varepsilon_{i}(t-d_{E})[\delta_{j,S}+\delta_{j,D}+\delta_{j,A}+\delta_{j,U}]\vartheta_{b}\circ P_{i}(j,t_{T},k,t_{k},l_{mat},l,t_{l},m,t_{m},n_{mat},n,t_{n},a,t)+$ |
| **7** |  |
| **8** | $\begin{matrix} \left[r_{B}k\frac{\partial}{\partial k}+r_{CA}l\frac{\partial}{\partial l}+r_{M}l_{mat}\frac{\partial}{\partial l}_{mat}+r_{ID}m\frac{\partial}{\partial m}+r_{M}n_{mat}\frac{\partial}{\partial n}_{mat}+r_{VA}n\frac{\partial}{\partial n}\right] \\ P_{i}(j,t_{T},k,t_{k},l_{mat},l,t_{l},m,t_{m},n_{mat},n,t_{n},a,t)+ \end{matrix}$ |
| **9** | $\begin{matrix} \mu(a)\delta(a)\delta(t_{T}+T_{big})\delta(t_{k}+T_{big})\delta(t_{l}+T_{big})\delta(t_{m}+T_{big}) \\ \delta(t_{n}+T_{big})\delta(l_{mat}-mat_{c}(\zeta_{i}))\delta(n_{mat}-mat_{v}(\zeta_{i})) \\ \delta_{i,S}\delta_{k,0}\delta_{l,0}\delta_{m,0}\delta_{n,0}P_{i}(j,t_{T},k,t_{k},l_{mat},l,t_{l},m,t_{m},n_{mat},n,t_{n},a,t)- \end{matrix}$ |
| **10** |  |

Lines 2-5 are the flows into the human compartments (T, U, D and A) shown in pathways represented in Fig. A. Line six represents the boosting of pre-erythrocytic immunity after exposure (in the case where exposure does not lead to an infection). Exposure that leads to infection of individuals in states S, A or U (with subsequent movement to states A, D or T) is shown in line seven. The communitive integral operators (defined below) track the density of individuals in each of the immune states. Deterministic exponential decay of the immunity types is represented in line 8. Line 9 and 10 represents birth and death processes.

Here $\vartheta_{I}$, $\vartheta_{b}$, $\vartheta_{c}$,$\vartheta_{d}$and$\vartheta_{v}$ are commutative integral operators with the following action on a density $f(j,t_{T},k,t_{k},l_{mat},l,t_{l},m,t_{m}n_{mat},n,t_{n},a,t)$:

$$\begin{matrix} \vartheta_{b}\circ f=\delta(t-t_{k})\int_{0}^{\infty} f(j,t_{T},k-1,t-u_{b}-\tau,l_{mat},l,t_{l},m,t_{m},n_{mat},n,t_{n},a,t)d\tau+ \Phi\left(\frac{t-t_{k}}{u_{b}}\right)f(j,t_{T},k,t_{k},l_{mat},l,t_{l},m,t_{m},n_{mat},n,t_{n},a,t) \\ \vartheta_{c}\circ f=\delta(t-t_{l})\int_{0}^{\infty} f(j,t_{T},k,t_{k},l_{mat},l-1,t_{l}-u_{c}-\tau,m,t_{m},n_{mat},n,t_{n},a,t)d\tau+ \Phi\left(\frac{t-t_{l}}{u_{c}}\right)f(j,t_{T},k,t_{k},l_{mat},l,t_{l},m,t_{m},n_{mat},n,t_{n},a,t) \\ \vartheta_{d}\circ f=\delta(t-t_{m})\int_{0}^{\infty} f(j,t_{T},k,t_{k},l_{mat},l,t_{l},m-1,t_{m}-u_{d}-\tau,n_{mat},n,t_{n},a,t)d\tau+ \Phi\left(\frac{t-t_{m}}{u_{d}}\right)f(j,t_{T},k,t_{k},l_{mat},l,t_{l},m,t_{m},n_{mat},n,t_{n},a,t) \\ \vartheta_{v}\circ f=\delta(t-t_{n})\int_{0}^{\infty} f(j,t_{T},k,t_{k},l_{mat},l,t_{l},m,t_{m},n_{mat},n-1,t_{n}-u_{v}-\tau,a,t)d\tau+ \Phi\left(\frac{t-t_{n}}{u_{v}}\right)f(j,t_{T},k,t_{k},l_{mat},l,t_{l},m,t_{m},n_{mat},n,t_{n},a,t) \end{matrix}$$

where $\Phi(x)$ is an indicator function such that

$$\Phi(x)=\begin{matrix} 1 x<1 \\ 0 otherwise. \end{matrix}$$

For each individual, *k*, *l,* m and n are set to zero at birth while $t_{I},t_{T},t_{k},t_{l},t_{m},t_{n}$ are set to a large negative value $-T_{big}$ (to represent never having been exposed or infected). Each naturally-acquired immunity term increases by 1 for an individual whenever that individual receives an infectious bite (*k*), or is infected (l, m and n), if the previous boost to k, l, m and n occurred more than $u_{b},u_{c},u_{d}$ and $u_{v}$ days earlier, respectively. The maternal immunity levels $l_{mat}$ and $n_{mat}$are drawn at random from the equivalent immunity levels in an individual between aged 15 and 35 years in the same heterogeneity in exposure group $\xi_{i}$and are termed $mat_{c}(\zeta_{i})$and $mat_{v}(\zeta_{i})$for clinical and severe disease immunity respectively. These are not boosted by exposure. All immunity levels decay exponentially.

The infection-state dependent susceptibility to infection tracks the prophylactic period of the drug given at the last treatment and is defined as:

$$s_{i,j'}(t-t_{T})=\begin{matrix} 1 & j'=D,A,U \\ W(t-t_{T}) & j'=S \\ 0 & j'=T \end{matrix}$$

where $W(t-t_{T})$ is a Weibull survivorship function that decays over time and is specific to the ACT treatment.

### Text H. The Vector component: Model structure

The vector model is based on the deterministic model previously described in White et al (2011) [6], but is implemented in its equivalent compartmental stochastic form for adult mosquitoes. Upon hatching from eggs, larvae progress through early and late larvae stages (*E* and *L* compartments) before developing to the pupal stage $\left(P_{L}\right)$. The larval stages are regulated by density dependent mortality, with a time-varying carrying-capacity, $K$, that represents the ability of the environment to sustain breeding sites through different periods of the year and with the density of larvae in relation to the carrying-capacity regulated by a parameter $\gamma$. The carrying-capacity determines the mosquito density and hence the baseline transmission intensity in the absence of interventions.

We assume 50% of the emergent adult mosquitoes are female and all enter the susceptible state $\left(S_{M}\right)$. The rate at which adult female mosquitoes become infected is a function of the infectiousness of the human population including an appropriate time-lag $\left(t_{l}\right)$ to account for the period between humans becoming infected and becoming infectious. The force of infection towards mosquitoes $\left(\Lambda_{M}\right)$ is therefore dependent on all human infected states and is defined as:

$$\Lambda_{M}(t)=\frac{\alpha}{\omega}\int_{0}^{\infty} \int_{0}^{\infty} \zeta\psi(a)(c_{D}D(\zeta,a,t-t_{l})+c_{T}T(\zeta,a,t-t_{l})+c_{A}A(\zeta,a,t-t_{l})+c_{U}U(\zeta,a,t-t_{l})dad\zeta$$

where $\alpha$ is the biting rate on humans

$$\alpha=Q_{0}/\delta,$$

$Q_{0}$ quantifies the level of anthropophagy and $\delta$ is the mean time between feeds. The parameter $\omega$ represents a normalising constant for the biting rate over all ages:

$$\omega=\int_{0}^{\infty} \psi(a)\mathrm{g}(\mathrm{a})\mathrm{da}$$

where $g(a)$ is the human age distribution. There is a fixed delay $\tau_{M}$ before female mosquitoes become infectious to humans ($I_{M}$) and are then assumed to remain infectious until their death.

### Text I. Stochastic equations

The vector component of the model is structured in a compartmental rather than individual format with compartments $\left(E,L,P_{L},S_{M},I_{M}\right)$ denoting the number of larvae/pupae/adult female mosquitoes in the respective states. The forward equations for the system are then given by:

$$\begin{matrix} \frac{dP(E,L,P_{L},S_{M},I_{M},t)}{dt}= \\ \beta M(t)\left[P(E-1,L,P_{L},S_{M},I_{M},t)-P(E,L,P_{L},S_{M},I_{M},t)\right] \\ +\mu_{E}\left[\left(1+\frac{E+L+1}{K}\right)P(E+1,L,P_{L},S_{M},I_{M},t)-\left(1+\frac{E+L}{K}\right)P(E,L,P_{L},S_{M},I_{M},t)\right] \\ +r_{EL}\left[P(E+1,L-1,P_{L},S_{M},I_{M},t)-P(E,L,P_{L},S_{M},I_{M},t)\right] \\ +\mu_{L}\left[\left(1+\gamma\frac{E+L+1}{K}\right)P(E,L+1,P_{L},S_{M},I_{M},t)-\left(1+\gamma\frac{E+L}{K}\right)P(E,L,P_{L},S_{M},I_{M},t)\right] \\ +r_{L}\left[P(E,L+1,P_{L}-1,S_{M},I_{M},t)-P(E,L,P_{L},S_{M},I_{M},t)\right] \\ +\mu_{P}\left[P(E,L,P_{L}+1,S_{M},I_{M},t)-P(E,L,P_{L},S_{M},I_{M},t)\right] \\ +r_{PL}\left[0.5P(E,L,P_{L}+1,S_{M}-1,I_{M},t)+0.5P(E,L,P_{L}+1,S_{M},I_{M},t)-P(E,L,P_{L},S_{M},I_{M},t)\right] \\ +\mu_{M}\left[P(E,L,P_{L},S_{M}+1,I_{M},t)-P(E,L,P_{L},S_{M},I_{M},t)\right] \\ +\Lambda_{M}(t-\tau_{M})\left[P(E,L,P_{L},S_{M}+1,I_{M}-1,t)-P(E,L,P_{L},S_{M},I_{M},t)\right] \\ +\mu_{M}\left[P(E,L,P_{L},S_{M},I_{M}+1,t)-P(E,L,P_{L},S_{M},I_{M},t)\right] \end{matrix}$$

where $M(t)=S_{M}(t)+I_{M}(t)$ and $r_{EL}$, $r_{L}$ and $r_{PL}$ are the rates of progression from $E\to L$, $L\to P$ and $P\to S_{M}$ respectively.

## Text J. Parameter values

Parameter estimates presented here (Table B) are obtained from a number of previous publications and associated model fitting [1,3–5].

Table B. Model parameter values

| Parameter | Symbol | Estimate |
| --- | --- | --- |
| Human infection duration (days) |  |  |
| Latent period | $d_{E}$ | 12 |
| Patent infection | $d_{A}$ | 195 |
| Clinical disease (treated) | $d_{T}$ | 5 |
| Clinical disease (untreated) | $d_{D}$ | 5 |
| Sub-patent infection | $d_{U}$ | 110 |
| Treatment Parameters |  |  |
| Probability of seeking treatment if clinically diseased | $f_{T}$ | Variable |
| Age and heterogeneity |  |  |
| Age-dependent biting parameter | $\rho$ | 0.85 |
| Age-dependent biting parameter | $a_{0}$ | 8 years |
| Variance of the log heterogeneity in biting rates | $\sigma^{2}$ | 1.67 |
| Immunity reducing probability of infection |  |  |
| Maximum probability due to no immunity | $b_{0}$ | 0.590076 |
| Maximum relative reduction due to immunity | $b_{1}$ | 0.5 |
| Inverse of decay rate | $d_{B}$ | 10 years |
| Scale parameter | $I_{B0}$ | 43.8787 |
| Shape parameter | $\kappa_{B}$ | 2.15506 |
| Duration in which immunity is not boosted | $u_{B}$ | 7.19919 days |
| New-born immunity relative to mother’s | $P_{CM}$ | 0.774368 |
| Immunity reducing probability of clinical disease |  |  |
| Maximum probability due to no immunity |  | 0.791666 |
| Maximum relative reduction due to immunity |  | 0.000737 |
| Inverse of decay rate | $d_{CA}$ | 30 years |
| Scale parameter | $I_{C0}$ | 18.02366 |
| Shape parameter | $\kappa_{C}$ | 2.36949 |
| Duration in which immunity is not boosted | $u_{C}$ | 6.06349 days |
| Inverse of decay rate of maternal immunity | $d_{M}$ | 67.6952 days |
| Immunity reducing probability of detection |  |  |
| Minimum probability due to maximum immunity | $d_{1}$ | 0.160527 |
| Inverse of decay rate | $d_{ID}$ | 10 years |
| Scale parameter | $I_{D0}$ | 1.577533 |
| Shape parameter | $\kappa_{D}$ | 0.476614 |
| Duration in which immunity is not boosted | $u_{D}$ | 9.44512 days |
| Scale parameter relating age to immunity | $a_{D}$ | 21.9 years |
| Time-scale at which immunity changes with age | $f_{D0}$ | 0.007055 |
| Shape parameter relating age to immunity | $\gamma_{D}$ | 4.8183 |
| Immunity reducing probability of severe disease and mortality |  |  |
| Maximum probability due to no immunity | $\theta_{0}$ | 0.0749886 |
| Maximum relative reduction due to immunity | $\theta_{1}$ | 0.0001191 |
| Scale parameter | $I_{V0}$ | 1.09629 |
| Shape parameter | $\kappa_{V}$ | 2.00048 |
| Inverse of decay rate | $d_{VA}$ | 30 years |
| Duration in which immunity is not boosted | $u_{V}$ | 11.4321 days |
| Inverse of decay rate of maternal immunity | $d_{VM}$ | 76.8365 days |
| New-born immunity relative to mother’s | $P_{VM}$ | 0.195768 |
| Reduced probability of death due to treatment | $f_{VT}$ | 0.5 |
| Age-dependent severe disease risk modifier parameter | $f_{V0}$ | 0.141195 |
| Age-dependent severe disease risk modifier parameter | $a_{V}$ | 2493.41 |
| Age-dependent severe disease risk modifier parameter | $\gamma_{V}$ | 2.91282 |
| Mortality scaling factor from severe disease | $\nu$ | 0.065 |
| Infectiousness to mosquitoes |  |  |
| Lag from parasites to infectious gametocytes | $d_{g}$ | 12 days |
| Untreated disease | $c_{D}$ | 0.068 |
| Treated disease | $c_{T}$ | 0.021896 |
| Sub-patent infection | $c_{U}$ | 0.00062 |
| Parameter for infectiousness of state *A* | $\gamma_{1}$ | 1.82425 |
| Mosquito Population Model |  |  |
| Daily mortality of adults with no interventions | $\mu_{M}$ | Varies by spp |
| Mean time between feeds | $\delta$ | 3 days |
| Extrinsic incubation period | $d_{EM}$ | 10 days |
| Larval model |  |  |
| Average number of eggs laid per female mosquito per day | $\beta$ | 21.2/day |
| Early instar larval developmental period | $d_{E}$ | 6.64 days |
| Late instar developmental period | $d_{L}$ | 3.72 days |
| Pupal developmental period | $d_{P}$ | 0.643 days |
| Mortality rate of early-stage larvae (density dependent) | $\mu_{E}$ | 0.0338/day |
| Mortality rate of late-stage larvae (density dependent) | $\mu_{L}$ | 0.0348/day |
| Mortality rate of pupae (density independent) | $\mu_{P}$ | 0.249/day |
| Effect of density dependence on late instars relative to early instars | $\gamma$ | 13.25 |

## Text K. Vector Bionomics

Within Africa the relative abundance of the two dominant vector species (*Anopheles gambiae* and *An. arabiensis*) were identified using rainfall and humidity data [8]. In some cases a third species (*An. funestus*) was also considered (Table B). For countries outside of Africa dominant vector species were identified using presence absence data [9–12]. The relative proportions of these species was 0.5, 0.3, 0.2 for three species, 0.6, 0.4 for two species and 1 for one species. Characterising bionomics parameters for the African vector species are shown in table C. For species outside of Africa vectors were characterised into “high” or “low” profiles base on threshold values for behavioural traits, the bionomics for which as also shown in table C.

**Table C. Vector bionomics parameters** [1]

| Bionomics trait | *An gambiae s.s* | *An arabiensis* | *An funestus* | *“*High*”* | “Low’ |
| --- | --- | --- | --- | --- | --- |
| Anthropophagy | 0.92 | 0.71 | 0.94 | 0.90 | 0.60 |
| Endophily | 0.81 | 0.42 | 0.81 | 0.85 | 0.30 |
| % bites indoors | 0.97 | 0.96 | 0.98 | 0.96 | 0.60 |
| % bites indoors and in bed | 0.89 | 0.90 | 0.90 | 0.90 | 0.50 |

## Text L. Seasonality

Seasonality is incorporated in the model by allowing a time-varying carrying capacity whereby the carrying capacity of the environment to support mosquito larvae is

$$K(t)=K_{0}\frac{R\left(t\right)}{\overline{R}}$$

where $K_{0}$ is the carrying capacity, $\overline{R}$ the mean rainfall overt the year and $R\left(t\right)$, the time varying seasonal curve, estimated from rainfall data:

$R\left(t\right)=g_{0}+\sum_{i=1}^{3} g_{i}\cos(2\pi ti)+h_{i}\sin(2\pi ti)$,

representing the first three frequencies of a Fourier transform fit to rainfall estimates from the US Climate Prediction Center [13,14] for sub-Saharan Africa between 2002 and 2009, where $g$ and $h$ are fitted parameters. Elsewhere, a perennial seasonal profile was assumed (Table B).

## Text M. Endemicity

Estimates of malaria parasite prevalence in 2-10 year olds in 2015 [15] were used in conjunction with increases in coverage of interventions over time to estimate the baseline parasite prevalence in 2000 in Africa. Elsewhere the spatial limits of malaria transmission were based on parasite prevalence maps for 2010 [16] with the levels of parasite prevalence re-scaled to match WHO reported cases for WHO estimated cases for 2015. Population data were compiled from the Gridded Population of the World (GWPv4) datasets (2000-2020) [17], and adjusted to be consistent with UN estimates of country populations. Populations at risk were estimated by masking regions designated as outside of the spatial limits of P. falciparum [16].

## Text N. Treatment

Successful treatment acts to return a clinically infected individual to the susceptible class providing them a drug-dependent period of partial protection from infection. We assume that non-ACTs have a 75% probability of being effective in clearing an infection, whereas ACTs have a 95% probability of being effective. For ACTs, protection from infection was characterised using a a previously published pharmacokinetic-pharmacodynamic (PKPD) model, fitted to clinical trial data from six different sites in sub-Saharan Africa [6]. The protection from infection at time $u$ after effective treatment is denoted by $P_{T}(u)$, and the probability of reinfection is multiplied by $1-P_{T}(u)$ relative to the probability with no prophylaxis. The overall degree of protection can be quantified by the area under the curve

$$A_{T}=\int_{0}^{\infty} P_{T}(u) du$$

For first line treatment (Artemether-Lumefantrine) $A_{T}$ varies from 7 to 16 days depending on age. For historical treatment (Sulphadoxine-Pyrimethamine) $A_{T}$ is taken as 25 days, using a Weibull survivor function for $P_{T}(u)$ instead of a PKPD model

## Text O. LLINs and IRS

The action of LLINs and IRS are modelled following Griffin *et al* (2010) [2] with the addition of one modification that accounts for the possibility of an mosquito being killed by IRS prior to feeding [1]. The interventions are modelled probabilistically based with potential outcomes being that a female mosquito attempting to feed is repelled, killed or successful in its endeavour. The modified details from [2] are repeated here for ease of interpretation.

The probability that a blood-seeking mosquito successfully feeds will depend on the bionomics and behaviours of the mosquito (which are species dependent) and the anti-vectorial interventions present in the human population. There are 6 different outcomes of a mosquito attempting to feed:

1. It bites a non-human host
2. It is killed by the LLIN before it bites
3. It is killed by IRS before it bites
4. It is killed by IRS after it bites
5. It successfully feeds and survives that feeding attempt
6. It is repelled without feeding, either through the actions of LLIN or IRS.

Repelled mosquitoes attempt to find alternative blood meal sources (a process referred to as repeating). It is assumed that all livestock are kept outside of the house and therefore all mosquitoes that enter the house attempt to bite humans.

Assume that person *i* is protected by a given LLIN/IRS efficacy. Let the probability that a mosquito of a given species bites host *i* during a single attempt be $y_{i}$; the probability that a mosquito bites a host and survives the feeding attempt be $w_{i}$, and the probability that it is repelled without feeding be $z_{i}$. These probabilities exclude natural vector mortality, so that for an individual with no protection, $y_{i}=w_{i}=1$ and $z_{i}=0$.

During a single feeding attempt (which may be on animals or humans), the probability that a mosquito successfully feeds is:

$W=(1-Q_{0})+Q_{0}\sum_{i} \pi_{i}w_{i}$,

and is repelled without feeding with probability:

$Z=Q_{0}\sum_{i} \pi_{i}z_{i}$,

where in both equations $Q_{0}$is the proportion of bites taken on humans in the absence of any intervention and $\pi_{i}$ is the proportion of bites on humans that person *i* receives in the absence of any intervention.

The length of time spent looking for a blood meal and resting between feeds are $\delta_{1}$ and $\delta_{2}$ respectively. The mosquito feeding rate $f_{R}$ is given by $f_{R}=\frac{1}{\delta_{1}+ \delta_{2}}$. Parameter $\delta_{2}$ is assumed to be unaffected by the interventions, whilst $\delta_{1}$ is increased to $\delta_{1}=\frac{\delta_{10}}{1-Z}$ where $\delta_{10}$ is the value with no interventions.

The probabilities of surviving the periods of feeding and resting are $p_{1}$ and $p_{2}$*.* With no interventions,

$p_{10}=\exp(-\mu_{0}\delta_{10}), p_{2}=\exp(-\mu_{0}\delta_{2})$,

where $\mu_{0}$is the natural death rate. With interventions $p_{2}$is unchanged and

$$p_{1}= \frac{p_{10}W}{1-Zp_{10}}$$

The probability of surviving one feeding cycle is $p_{1}p_{2}$. Hence the mosquito death rate $\mu_{M}$can be found as,

$$\begin{matrix} p_{1}p_{2}=\exp(-\mu/f_{R}) \\ \\ \mu=-f_{R}\log(p_{1}p_{2}). \end{matrix}$$

The probability of surviving the extrinsic incubation period, $P_{M}$, therefore also changes as* *$\mu$ changes.

The probability that a feeding cycle ends with a successful bite on person *i* ,$q_{i}$, is,

$$\begin{matrix} q_{i}=p_{10}\left(Q_{0}\pi_{i}w_{i}+Zq_{i}\right) \\ q_{i}=\frac{p_{10}Q_{0}\pi_{i}w_{i}}{1-Zp_{10}} \end{matrix}$$

The probability that a feeding cycle ends with a bite on an animal is,

$$\begin{matrix} q_{A}=p_{10}\left(1-Q_{0}+Zq_{A}\right) \\ q_{A}=\frac{p_{10}(1-Q_{0})}{1-Zp_{10}} \end{matrix}$$

Hence the proportion of successful bites which are on humans is,

$$\begin{matrix} Q=1-\frac{q_{A}}{q_{A}+\sum_{i} q_{i}}=1-\frac{(1-Q_{0})}{(1-Q_{0})+Q_{0}\sum_{i} \pi_{i}w_{i}} \\ =1-\frac{(1-Q_{0})}{W} \end{matrix}$$

and the biting rate on humans is,

$\alpha=Qf_{R}$ .

The rate at which person *i* is bitten by this species is,

$\lambda_{i}=\frac{\alpha\pi_{i}w_{i}}{\sum_{i} \pi_{i}w_{i}}$,

When IRS is used, some mosquitoes may bite a person before dying by picking up a lethal insecticide dose when resting on the walls of the house. So for calculating the force of infection on humans, the biting rate on each person needs to be inflated by a factor $\frac{y_{i}}{w_{i}}$giving an effective biting rate when estimating EIR of,

$$\tilde{\lambda}_{i}=\frac{\alpha\pi_{i}y_{i}}{\sum_{i} \pi_{i}w_{i}}$$

The EIR experienced by person *i* due to this mosquito species is $\tilde{\lambda}_{i}I_{M}$ and the total EIR they experience is the sum EIRs, attributatble to each vector species present.

### Text P. Vector outcomes

The degree of protection afforded by LLIN and IRS will depend on the proportion of bites humans receive while protected by the intervention [2,3]. This will depend on host movement / sleeping patterns, the biting behaviour of the mosquito vector and the efficacy of the intervention.

Let the rate at which a person who is indoors at hour *t* is bitten be $\lambda_{I}(t)$ , and the corresponding figure for someone outdoors be $\lambda_{O}(t)$. Knowing the proportion of human hosts indoors $p_{I}(t)$ or in bed $p_{B}(t)$ at a given time *t* enables us to calculate the proportion of bites taken on humans while they are indoors as,

$\Phi_{I}=\frac{\sum_{t} p_{I}(t)\lambda_{I}(t)}{\sum_{t} \left((1-p_{I}(t))\lambda_{O}(t)+p_{I}(t)\lambda_{I}(t)\right)}$ ,

whereas the proportion of bites taken on the human population while they are in bed is,

$\Phi_{B}=\frac{\sum_{t} p_{B}(t)\lambda_{I}(t)}{\sum_{t} \left((1-p_{I}(t))\lambda_{O}(t)+p_{I}(t)\lambda_{I}(t)\right)}$.

Due to the lack of data it is assumed that human movement and sleeping patterns are not dependent on age or relative exposure.

Once a mosquito enters a house to feed, one of three things can happen: it can repeat, feed successfully or die. shows the order in which the different processes operate when a mosquito attempts to feed on a person protected with both LLIN and IRS.


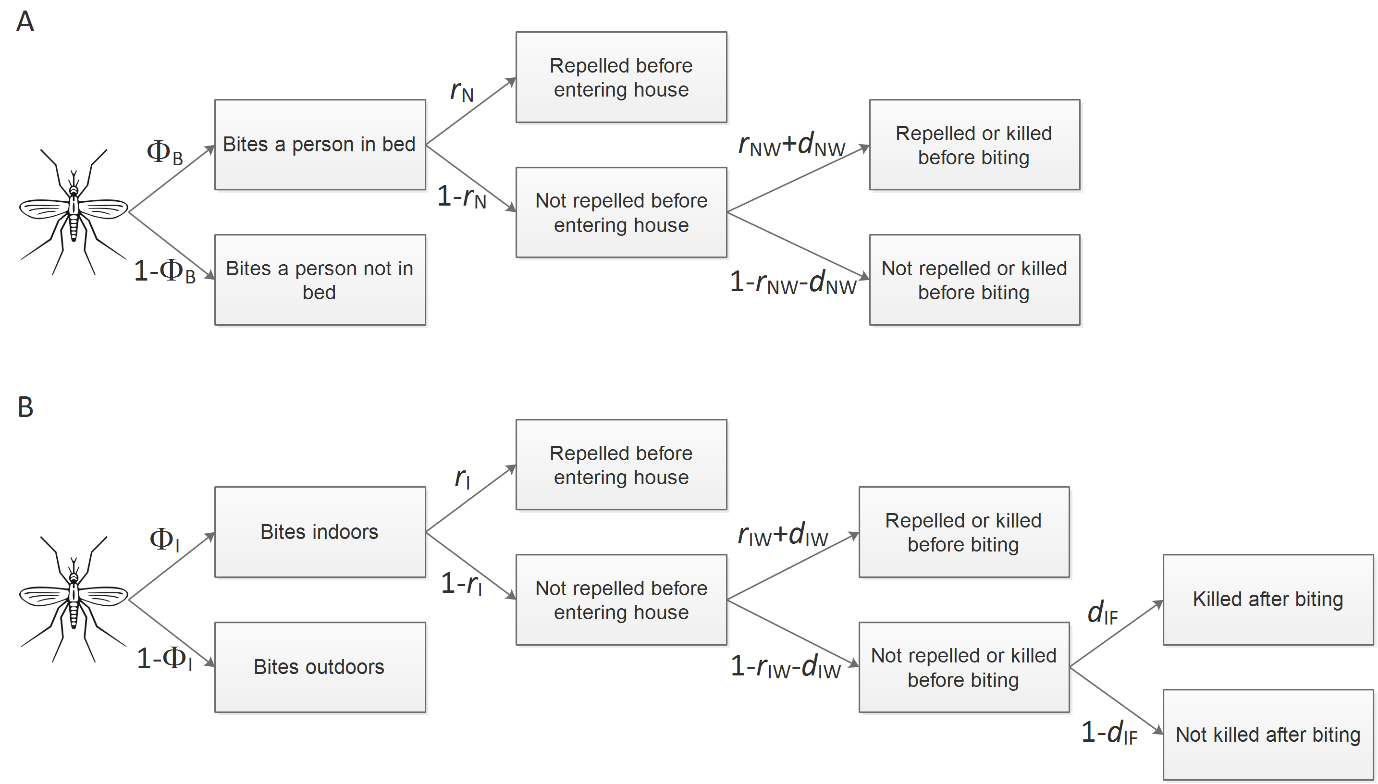


Figure B Probability tree for mosquitoes attempting to feed in the presence of A) LLINs and B) IRS.

The probabilities of each outcome, namely successful feeding, any biting and repulsion, for a feeding attempt on a human are shown in fig. B and table D.

**Table D. Vector control probabilistic model.** Outcomes are probabilities for vector feeding, biting and repulsion in the presence of vector control.

| Outcome | Probability |
| --- | --- |
| Successful feed (*w_i_*) |  |
| IRS only | $1-\Phi_{I}+\Phi_{I}(1-r_{I})(1-r_{IW}-d_{IW})(1-d_{IF})$ |
| LLIN only | $1-\Phi_{B}+\Phi_{B}(1-r_{N})(1-r_{NW}-d_{NW})$ |
| IRS plus LLIN | $\begin{matrix} 1-\Phi_{I}+(\Phi_{I}-\Phi_{B})(1-r_{I})(1-r_{IW}-d_{IW})(1-d_{IF}) \\ \Phi_{ B}(1-r_{I})(1-r_{IW}-d_{IW})(1-d_{IF})(1-r_{N})(1-r_{NW}-d_{NW}) \end{matrix}$ |
| Biting (*y_i_*) |  |
| IRS only | $1-\Phi_{I}+\Phi_{I}(1-r_{I})(1-r_{IW}-d_{IW})$ |
| LLIN only | $1-\Phi_{B}+\Phi_{B}(1-r_{N})(1-r_{NW}-d_{NW})$ |
| IRS plus LLIN | $\begin{matrix} 1-\Phi_{I}+(\Phi_{I}-\Phi_{B})(1-r_{I})(1-r_{IW}-d_{IW}) \\ \Phi_{ B}(1-r_{I})(1-r_{IW}-d_{IW})(1-r_{N})(1-r_{NW}-d_{NW}) \end{matrix}$ |
| Repulsion (*z_i_*) |  |
| IRS only | $\Phi_{I}\left(r_{I}+(1-r_{I})r_{IW}\right)$ |
| LLIN only | $\Phi_{B}\left(r_{N}+(1-r_{N})r_{NW}\right)$ |
| IRS plus LLIN | $\left(\Phi_{I}-\Phi_{B}\right)\left(r_{I}+(1-r_{I})r_{IW}\right)+\Phi_{B}\left(r_{I}+(1-r_{I})\left(r_{N}+(1-r_{N})(r_{IW}+(1-r_{IW}-d_{IW})r_{NW}\right)\right)$ |

where $\Phi_{I}$ and $\Phi_{B}$ represent the probabilities (with respect to the time of day) of feeding indoors and on someone in bed respectively, $r_{N}$ and $r_{I}$ represent the probability of being repelled before entering the house due to LLINs and IRS respectively, $r_{NW}$ the probability of being repelled by the bednet and $d_{NW}$ the probability of being killed by the bednet after entering the house. The parameters $r_{IW}$, $d_{IW}$ and $d_{IF}$ are the probabilities of being repelled before feeding, killed before feeding or killed after feeding after entry to a house due to IRS, where $d_{IF}$ is species specific.

The parameter values used for the probability model are summarised in table E.

**Table E. Vector control parameters** [1]

| Intervention | Probability | Symbol | Value |
| --- | --- | --- | --- |
| LLINs | Repelled before entering the house | $r_{N}$ | 0.113 |
|  | Repelled by the bednet | $r_{NW}$ | 0.295 |
|  | Killed by the bednet | $d_{NW}$ | 0.533 |
| IRS | Repelled before entering the house | $r_{I}$ | 0.687 |
|  | Repelled by the IRS | $r_{IW}$ | 0 |
|  | Killed before feeding | $d_{IW}$ | 0.295 |
|  | Killed after feeding (An. gambiae ss and funestus) | $d_{IF}$ | 0.813 |
|  | Killed after feeding (An. arabiensis) | $d_{IF}$ | 0.422 |

## Text Q. SMC

Seasonal malaria chemoprevention (SMC) consisted of 3 courses of drug (Sulfadoxine/pyrimethamine + amodiaquine) given to children between 6 months and 5 years of age during the transmission season. Time of the treatment coincides with the peak season, as defined by the maximum carrying capacity, with the first treatment occurring one month prior and the last treatment one month post the seasonal peak.

The action of SMC as implemented in the model is assumed to be identical to treatment in that those infected and receiving the drug have a probability of successfully clearing infection and moving into the susceptible disease state and all those that clear infection or were in the susceptible state receive a period of drug-dependent partial protection from infection prophylaxis [1,6]. Protection from reinfection following each course of treatment is parameterised using a Weibull survival curve as in section 2.1.

# Text R. Estimating DALYs

DALY calculation followed guidelines from the global burden of disease study [20]. DALYS consist of the sum of two components, Years of Life Lost (YLL) and Years of Life with Disease (YLD). YLL is calculated using estimates of the number of deaths and a life expectancy of 56 (estimated for all non-high income sub-Saharan African countries [21]).

YLD is calculated using estimates of the number of clinical and severe cases of disease in each age group combined with the length of an episodes of clinical or severe malaria and associated weighting for the severity of disease (Table F).

Table F. Disability adjusted life year parameters [20]

| Disease manifestation | Length of episode ($L$) | Disability weight ($W$) |
| --- | --- | --- |
| Uncomplicated (0-5) | 0.01375 | 0.211 |
| Uncomplicated (5-15) | 0.01375 | 0.195 |
| Uncomplicated (15-99) | 0.01375 | 0.172 |
| Severe | 0.04795 | 0.600 |

**Table G. Country-level projections of the estimated additional cases and deaths in the period 2017 -2020 due to a 44% cut in PMI-funding.**

| Country | Additional cases (millions) (95% CrI) | Additional Deaths (thousands) (95% CrI) |
| --- | --- | --- |
| Angola | 2.0 (1.6, 2.6) | 11.3 (6.4, 18.4) |
| Burundi | 0.1 (0, 0.2) | 0.4 (0.1, 0.8) |
| Benin | 2.7 (2.0, 3.2) | 9.8 (5.9, 14.0) |
| Burkina Faso | 0.3 (0, 0.6) | 0.3 (-0.1, 1.6) |
| Cambodia | 0.0 (0.0, 0.0) | 0.0 (0.0, 0.1) |
| DRC | 3.5 (2.0, 5.9) | 29.8 (16.5, 41.6) |
| Ethiopia | 2.1 (0.9, 3.1) | 9.2 (4.1, 16.0) |
| Ghana | 3.4 (2.2, 4.8) | 17.2 (9.5, 22.2) |
| Guinea | 0.7 (0.5, 1.1) | 6.8 (4.5, 14) |
| Kenya | 4.4 (3.2, 5.6) | 22.6 (12.1, 34) |
| Liberia | 0.6 (0.4, 0.9) | 3.5 (2, 5.1) |
| Madagascar | 0.6 (0.4, 0.9) | 3.7 (2.2, 5.8) |
| Malawi | 1.4 (1, 2) | 9.2 (5, 12.8) |
| Mali | 5.5 (4.7, 6.6) | 17.1 (10, 34.2) |
| Mozambique | 5.5 (4.1, 7) | 20.3 (12.2, 27) |
| Myanmar | 0.1 (-0.1, 0.3) | 0.6 (-0.4, 1.9) |
| Nigeria | 21.9 (14.6, 28.2) | 78.7 (44.6, 107.4) |
| Rwanda | 1.4 (1.1, 1.6) | 6 (3.1, 10.3) |
| Senegal | 0.8 (0.7, 1) | 4.1 (2, 6.7) |
| South Sudan | 0.6 (0.3, 1) | 1.2 (0.5, 2.1) |
| Tanzania | 3.1 (1.8, 4.4) | 17.5 (8.9, 28.5) |
| Uganda | 3.4 (2.8, 3.8) | 9.2 (15.2, 12.2) |
| Zambia | 2.4 (2.1, 2.6) | 8.8 (5.1, 12.6) |
| Zimbabwe | 0.2 (0.2, 0.4) | 1.9 (1, 2.8) |
| Total/Summary | 82.5 (61.3, 98.3) | 331.7 (190.7, 450.4) |

# References

1. Griffin JT, Bhatt S, Sinka ME, Gething PW, Lynch M, Patouillard E, et al. Potential for reduction of burden and local elimination of malaria by reducing Plasmodium falciparum malaria transmission: a mathematical modelling study. Lancet Infect Dis. Griffin et al. Open Access article distributed under the terms of CC BY; 2016;3099: 1–8. doi:10.1016/S1473-3099(15)00423-5

2. Griffin JT, Hollingsworth TD, Okell LC, Churcher TS, White M, Hinsley W, et al. Reducing Plasmodium falciparum malaria transmission in Africa: a model-based evaluation of intervention strategies. Krishna S, editor. PLoS Med. Public Library of Science; 2010;7: e1000324. doi:10.1371/journal.pmed.1000324

3. Griffin JT, Ferguson NM, Ghani AC. Estimates of the changing age-burden of Plasmodium falciparum malaria disease in sub-Saharan Africa. Nat Commun. Nature Publishing Group; 2014;5: 3136. doi:10.1038/ncomms4136

4. White MT, Griffin JT, Churcher TS, Ferguson NM, Basáñez MG, Ghani AC. Modelling the impact of vector control interventions on Anopheles gambiae population dynamics. Parasit Vectors. 2011;4: 153. doi:10.1186/1756-3305-4-153

5. Griffin JT, Hollingsworth TD, Reyburn H, Drakeley CJ, Riley EM, Ghani AC. Gradual acquisition of immunity to severe malaria with increasing exposure. Proc Biol Sci. 2015;282. doi:10.1098/rspb.2014.2657

6. Okell LC, Cairns M, Griffin JT, Ferguson NM, Tarning J, Jagoe G, et al. Contrasting benefits of different artemisinin combination therapies as first-line malaria treatments using model-based cost-effectiveness analysis. Nat Commun. Nature Publishing Group; 2014;5: 5606. doi:10.1038/ncomms6606

7. United Nations. Population Trends [Internet]. [cited 7 Jun 2017]. Available: http://www.un.org/en/development/desa/population/theme/trends/index.shtml

8. Lindsay SW, Parson L, Thomas CJ. Mapping the ranges and relative abundance of two principal African malaria vectors, Anopheles gambiae sensu stricto and An. arabieasis, using climate data. Proc R Soc London, Ser B. 1998;265: 847–854.

9. Sinka ME, Bangs MJ, Manguin S, Coetzee M, Mbogo CM, Hemingway J, et al. The dominant Anopheles vectors of human malaria in Africa, Europe and the Middle East: occurrence data, distribution maps and bionomic précis. Parasit Vectors. BioMed Central Ltd; 2010;3: 117. doi:10.1186/1756-3305-3-117

10. Sinka ME, Bangs MJ, Manguin S, Chareonviriyaphap T, Patil AP, Temperley WH, et al. The dominant Anopheles vectors of human malaria in the Asia-Pacific region: occurrence data, distribution maps and bionomic précis. Parasit Vectors. BioMed Central Ltd; 2011;4: 89. doi:10.1186/1756-3305-4-89

11. Sinka ME, Bangs MJ, Manguin S, Rubio-Palis Y, Chareonviriyaphap T, Coetzee M, et al. A global map of dominant malaria vectors. Parasit Vectors. 2012;5: 69. doi:10.1186/1756-3305-5-69

12. Sinka ME, Bangs MJ, Manguin S, Chareonviriyaphap T, Patil AP, Temperley WH, et al. The dominant Anopheles vectors of human malaria in the Americas: occurrence occurrence data, distribution maps and bionomic précis. Parasit Vectors. 2011;4: 89. doi:10.1186/1756-3305-4-89

13. National Weather Service. Climate Prediction Center [Internet]. [cited 24 Mar 2016]. Available: http://www.cpc.ncep.noaa.gov/products/international/

14. Garske T, Ferguson NM, Ghani AC. Estimating Air Temperature and Its Influence on Malaria Transmission across Africa. PLoS One. 2013;8. doi:10.1371/journal.pone.0056487

15. Bhatt S, Weiss DJ, Cameron E, Bisanzio D, Mappin B, Dalrymple U, et al. The effect of malaria control on Plasmodium falciparum in Africa between 2000 and 2015. Nature. 2015;8: 207–211. doi:10.1038/nature15535

16. Gething PW, Patil AP, Smith DL, Guerra C a., Elyazar IRF, Johnston GL, et al. A new world malaria map: Plasmodium falciparum endemicity in 2010. Malar J. 2011;10: 378. doi:10.1186/1475-2875-10-378

17. CIESIN. Gridded Population of the World (GPW), v4 [Internet]. 2016. Available: http://beta.sedac.ciesin.columbia.edu/data/collection/gpw-v4

18. World Health Organization. World Malaria Report. Geneva; 2016.

19. USAID. The President’s Malaria Initiative: Tenth Annual Report to Congress. 2016.

20. Murray CJL, Ezzati M, Flaxman AD, Lim S, Lozano R, Michaud C, et al. GBD 2010: Design, definitions, and metrics. Lancet. 2012;380: 2063–2066. doi:10.1016/S0140-6736(12)61899-6

21. The World Bank. Life expectancy at birth, total (years) [Internet]. Available: http://data.worldbank.org/indicator/SP.DYN.LE00.IN
